# Plastome phylogenomics unveils an East Asian origin and climatic niche-driven radiation of the temperate tribe Polygoneae (Polygonaceae)

**DOI:** 10.3389/fpls.2026.1792990

**Published:** 2026-03-18

**Authors:** Dong-Ling Cao, Yu-Ting Han, Yue Zhang, Jun-Qi Wang, Yan Xing, Xue-Jie Zhang, Shou-Jin Fan

**Affiliations:** College of Life Sciences, Shandong Normal University, Jinan, China

**Keywords:** Adaptive radiation, morphological homoplasy, niche evolution, plastome phylogenomics, Polygoneae

## Abstract

As a core lineage within Polygonoideae, the tribe Polygoneae holds significant taxonomic and evolutionary importance. These species are primarily distributed across the North Temperate Zone, with a few extending into the tropics. China serves as a distribution hotspot, particularly in its southwestern mountains and the arid regions of Xinjiang, hosting remarkable diversity that reflects the tribe’s extensive adaptive radiation in dry habitats. Despite its ecological prominence, the phylogenetic relationships and spatiotemporal diversification patterns of the tribe remain poorly resolved. We employed plastome phylogenomics to reconstruct a robust evolutionary framework for Polygoneae and allied taxa, with comprehensive sampling of 175 accessions representing 163 species across 21 genera of Polygonaceae. Phylogenetic analyses yielded a well-supported topology resolving previously contentious relationships. Molecular dating indicates that Polygoneae experienced an intense burst of diversification in East Asia during the late Oligocene (28–20 Ma), followed by a rapid radiation during the Miocene-Pliocene transition (8–3 Ma). These diversification phases show a temporal correspondence with the intensification of the East Asian monsoon and the uplift of the Tianshan Mountains. Ancestral area reconstruction confirmed an East Asian origin for Polygoneae, with subsequent dispersals across the Northern Hemisphere occurring through three major corridors. Specifically, the Southwest China-Central Asia corridor acted as an environmental filter, favoring the westward expansion of xeric-adapted woody shrubs; the Southwest-Northeast corridor served as a mesic refugium, maintaining stable conditions for herbaceous lineages throughout Quaternary glacial-interglacial cycles; and the Hengduan-Qinling-Yanshan mountain corridor facilitated liana dispersal via its topographically complex terrain. Ecological niche modeling demonstrated that climatic niche shifts, particularly adaptations to aridity and temperature extremes, drove lineage diversification. The trait evolution analyses revealed multiple independent origins of woodiness among arid-adapted genera, demonstrating convergent evolution in response to xeric habitats. By reconstructing a comprehensive phylogeny of Polygoneae using complete plastome sequences, this study reveals its East Asian origin and climate-driven radiation pattern. These findings highlight the pivotal role of Cenozoic geological and climatic changes in shaping temperate Asian plant diversity, offering insights into adaptive radiation mechanisms under environmental heterogeneity.

## Introduction

The Cenozoic India-Eurasia collision has fundamentally reshaped Eurasian landscapes and climate, driving extraordinary plant diversification (Cao et al., 2025; Barbolini et al., 2020). Cenozoic tectonic activity and climate oscillations across Eurasia have profoundly shaped plant biodiversity, with orogenic uplift events, such as the Neogene uplift of the Qinghai-Tibetan Plateau (QTP), acting as pivotal drivers of rapid speciation and endemic radiations (Li and Wang, 2020; Wu et al., 2022; Miao et al., 2022). The Tianshan Mountains in Xinjiang were rejuvenated, driven by the far-field effects of the India-Eurasia collision and the subsequent uplift of the Tibetan Plateau. As a result, they have become a significant center for plant diversification, particularly within the Polygonaceae (Zhang et al., 2014; Xu et al., 2016; Wu et al., 2022). Stretching approximately 2,500 km across Central Asia, this east-west mountain range features complex topography, including high-elevation plateaus (>4,000 m above sea level), deeply incised valleys, and localized microclimates, creating a fragmented landscape of ecological niches. The Tianshan has served as a dynamic arena for plant radiation, influenced by Pleistocene glacial-interglacial cycles that repeatedly contracted and expanded the distribution of habitats, as well as a long-term trend toward aridification driven by global cooling, intensified rain-shadow effects of the QTP, and the retreat of the Paratethys Sea (Zheng et al., 2025; Kaya et al., 2019). As the main ecological corridor in Xinjiang, the Tianshan Mountains provide critical habitats and function as a center of diversity for Polygoneae. The tribe Polygoneae, which comprises approximately 200 species exhibiting remarkable ecological adaptability, serves as an excellent model system for studying these biogeographic processes and the pervasive homoplasy driven by convergent adaptation (Schuster et al., 2011a; Cao et al., 2023). Polygoneae lineages span elevation and moisture gradients, ranging from alpine meadows and riparian zones to dry rocky slopes, and display convergent adaptations to extreme environmental stressors. Woody shrubs, such as *Atraphaxis* (Tavakkoli et al., 2014; Jafari et al., 2025), developed drought-tolerance features (such as sclerophyllous leaves and deep taproots) to colonize arid montane zones, while lianoid genera, such as *Fallopia*, evolved shade-adapted strategies (e.g., rapid stem elongation, helical tendrils) for humid forest understories (Wang et al., 2018; Zhang et al., 2022c). Herbaceous perennials like *Knorringia* dominate saline-alkali soils through genetic adaptations in ion transport and stem osmoregulation (Schuster et al., 2011b; Zhang et al., 2022b). Herbaceous annuals and perennials within *Polygonum* have convergently evolved a suite of xeromorphic adaptations to withstand the extreme diurnal temperature fluctuations and severe aridity of the region’s interior basins and rain-shadowed valleys. These adaptations, such as significantly reduced leaf area, suberized stems, and brachyblast development, enabled their survival in microrefugia during glacial maxima, thereby promoting genetic isolation and ultimately leading to peripatric speciation (Zhang et al., 2022c; Cao et al., 2023).

The systematics of the Polygoneae remain challenging to resolve, primarily because *Polygonum* is polyphyletic, largely due to widespread morphological homoplasy (Zhang et al., 2022c; Mahmoudi et al., 2020). Distantly related lineages have independently developed similar traits, such as woody stems and reduced floral parts, through convergent evolution in arid environments. These homoplastic traits, including growth habit (herbaceous versus woody) and perianth shape, obscure true phylogenetic relationships (Tavakkoli et al., 2014; Sun et al., 2014). Traditional classifications based on these variable characters have thus created artificial groupings, as evidenced by the polyphyly of *Polygonum* and the unclear boundaries between *Polygonum* and *Araphaxis* (Hong et al., 2005; Decraene and Smets, 2000). Molecular phylogenies often differ from those based solely on morphology, particularly in groups characterized by reticulate evolution, ecological convergence, and hybridization (Schuster et al., 2015). The ADP group (including *Atraphaxis*, *Duma*, and *Polygonum*) remains phylogenetically unstable due to limited sampling, widespread homoplasy in dry-adapted traits (e.g., convergent sclerophylly), and conflicting molecular signals from morphological versus plastid data (Tavakkoli et al., 2014; Schuster et al., 2013), as illustrated by the case of the woody plant *P. popovii*, which is endemic to the Tianshan Mountains in Xinjiang, China, and was originally classified under *Polygonum* due to its leaf and flower structure. However, its classification remains debated because it inhabits the same dry environments and shares xeric traits, such as stem lignification and small microphylls (≤5 mm²), which closely resemble those of *Atraphaxis* (Yurtseva et al., 2016; Zhang et al., 2014). The pervasive discordance between genomic compartments highlights the limitations of single-marker approaches. It underscores the necessity for genome-scale plastid data to clarify deep phylogenetic relationships and identify potential hybridization events. In combined plastid analyses (*trnL* + *trnL-F*, *rpl32-trnL*), *Duma* is consistently resolved as a weakly supported sister lineage to the clade, nested within the ADP clade. In stark contrast, nuclear ITS phylogenies based on MAFFT-aligned matrices consistently associate *Duma* with the RMF clade (*Reynoutria–Muehlenbeckia–Fallopia*), albeit with weak support, rather than placing it within the ADP clade (Schuster et al., 2011b; Zhang et al., 2022b). Molecular phylogenies reveal that the taxonomically challenging RMF group is paraphyletic, thus challenging the current genus boundaries (Schuster et al., 2011b; Desjardins et al., 2023). These generic boundaries were initially defined by morphological traits, including extrafloral nectaries at the petiole base, a liana habit, and papillate or fringed stigmas. However, phylogenetic studies consistently place some *Fallopia* species within *Reynoutria*, rendering *Reynoutria* paraphyletic (Schuster et al., 2011b, Schuster et al., 2015; Desjardins et al., 2023). Based on chloroplast genome analyses, phylogenetic studies reveal significant topological conflicts in *F. cynanchoides*. The chloroplast data consistently show problematic placement, with this species forming sister-group relationships with either *Rumex* or *Atraphaxis* across different reconstructions (Zhang et al., 2022c). Likewise, species such as *F. giraldii*, now classified as *Pteroxygonum giraldii* based on molecular and morphological differences, highlight previous misclassification caused by homoplasy (Schuster et al., 2015; Wang and Chen, 2020b; Cao et al., 2023). Morphological homoplasy, especially in traditional taxonomic characters like life form and perianth morphology, obscures phylogenetic reconstruction. The scarcity of genome-scale data and insufficient sampling of key xerophytic lineages, particularly those from Central Asian biodiversity hotspots like the Tianshan Mountains, have hindered the reconstruction of a robust phylogenetic framework. Such limitations have hindered a comprehensive understanding of the tribe’s evolutionary trajectory and adaptive diversification.

Recent phylogenomic analyses demonstrate that combining ecological niche data with molecular markers helps resolve persistent conflicts in taxonomically complex, reticulate systems. ENM (Gaynor et al., 2020), which merges species occurrence data with bioclimatic variables to reconstruct past distributions and examine niche dynamics, has become indispensable for biogeographic research, especially in regions with limited fossil records such as Central Asia (Lyu et al., 2024; Thompson et al., 2024). By integrating species occurrence data with climatic variables, ENM enables the reconstruction of paleodistributions, the identification of glacial refugia, and the forecasting of future range shifts (Tang et al., 2018; Jafari et al., 2025). For instance, ENM can test whether the arid-adapted subclade within Polygoneae in the Tianshan Mountains diverged due to Pleistocene glacial and interglacial habitat contractions or niche conservation during prolonged Miocene-Pliocene aridification (Xu and Zhang, 2015; Aitken et al., 2008). Here we use ecological niche modeling (ENM) within a robust phylogenetic framework to test between two competing hypotheses for Polygoneae: whether the distribution of arid-adapted lineages stems from niche conservatism during ancient (Miocene-Pliocene) aridification, or from more recent niche evolution in response to Pleistocene habitat fragmentation (Zhang et al., 2014; Song et al., 2021; Wang et al., 2007). By distinguishing true homoplasy from phylogenetic conservatism, comparative niche analyses provide a critical framework that offers direct insights into the adaptive pathways underpinning the tribe’s diversification.

Our study, along with existing phylogenies, reveals stark topological conflicts within Polygoneae, underscoring the necessity for an integrative taxonomic framework that synthesizes molecular, morphological, and ecological evidence. To address this, we employed plastid phylogenomics with extensive taxon sampling (163 species/175 individuals), strategically targeting underrepresented and ecologically critical lineages from East Asian and Central Asian biodiversity hotspots, including the Qinghai–Tibetan Plateau (QTP) and the Tianshan Mountains. Our sampling prioritized key Xinjiang endemics to capture the morphological and ecological diversity of the Polygoneae. This included the woody *P. popovii*, several rarely sampled *Polygonum* species (e.g., *P. tachengense* and *P. urumqiense*), and xerophytic genera such as *Atraphaxis*. Within a fossil-calibrated phylogenomic framework, we integrated divergence dating, ancestral state reconstruction, phylogenetic signal analysis of key traits, and tests for selection to explicitly investigate the roles of homoplasy and niche evolution in the tribe’s spatiotemporal diversification. Our study aims to: (1) reconstruct a robust phylogeny of Polygoneae using complete plastomes and trace the evolution of critical traits to differentiate synapomorphies from homoplastic adaptations; (2) estimate divergence times and link major speciation events to Cenozoic climatic shifts and orogenic activities, particularly the uplift of the QTP and the development of the East Asian monsoon; and (3) infer the historical migration routes and project their future habitat suitability under climate change scenarios.

## Materials and methods

### Taxon sampling

Our comprehensive sampling strategy for Polygoneae encompassed 175 accessions representing 163 species across 21 genera of Polygonaceae, with deliberate emphasis on capturing the tribe’s complete evolutionary and ecological diversity. Within Polygoneae, we sequenced 24 new plastomes and compiled a dataset of 50 species across all 10 recognized genera, achieving comprehensive representation of the tribe’s taxonomic breadth. To minimize taxonomic bias and ensure robust phylogenetic resolution, our sampling was designed to target four key categories of taxa:(1) ecologically significant lineages of Polygoneae, with complete genus-level coverage. This included arid-adapted genera such as *Atraphaxis* (5 species, all Central Asian endemics); (2) phylogenetically critical, narrowly distributed taxa that are essential for resolving tribal relationships. Notable examples are Xinjiang endemics (*Polygonum urumqiense*, *P. tachengense*) and the Tianshan-restricted *P. popovii*, all of which serve as key indicators of biogeographic patterns; (3) taxonomically complex groups requiring enhanced sampling to test monophyly. These included Polygonum sensu stricto (sampled species: *P. patulum*, *P. argyrocoleon*, *P. cognatum*) and *Fallopia* (7 species, 9 accessions), with sampling aimed at resolving the historically problematic ADP and RMF clades; (4) representatives from non-arid habitats (*Reynoutria*: 1 species, 3 accessions; *Polygonella americana*) to capture the full ecological spectrum of Polygoneae, from arid shrubs to forest linans. The chloroplast dataset comprised 94 newly sequenced plastomes from our field collections, 72 retrieved from GenBank, and 27 chloroplast fragments from 11 additional samples to strengthen phylogenetic resolution at key nodes. Geographic sampling focused on Polygoneae’s major diversification centers, the Qinghai-Tibetan Plateau, Tianshan Mountains, and Hengduan Mountains, ensuring representation of all major biodiversity hotspots that have shaped tribal evolution. This strategic over-representation of taxonomically complex lineages provides the resolution needed to test critical phylogenetic hypotheses while maintaining balanced coverage across herbaceous, woody, and liana life forms. All voucher specimens are deposited in the Shandong Normal University Herbarium (SDNU; **Supplementary Tables S1**, **S2**), with complete metadata, including collector codes, GPS coordinates, habitat descriptions, and NCBI accession numbers, to ensure full reproducibility.

### DNA extraction, sequencing, and assembly

High-quality genomic DNA was isolated from silica-gel-dried leaf tissues or herbarium specimens using a modified CTAB method, followed by RNase A treatment to remove RNA contamination. DNA integrity was assessed via 1% agarose gel electrophoresis, confirming the presence of clear, high-molecular-weight bands (>10 kb). Purity was evaluated using a NanoDrop 2000 spectrophotometer (Thermo Fisher Scientific), with acceptable ratios of A_260_/A_280_ between 1.8 - 2.0 and A_260_/A_230_ > 2.0. Sequencing libraries were constructed using the NEB Next^®^ Ultra^™^ DNA Library Prep Kit (New England Biolabs), with a target insert size of ~ 350 bp. Paired-end sequencing (2 × 150 bp) was conducted on an Illumina NovaSeq X Plus Series PE150 platform (Novogene Co., Ltd., Beijing, China). Approximately 5 Gb of raw data were generated per sample, with a minimum base quality score (Q30) exceeding 90%. Considering that chloroplast genomes typically constitute only 0.5% to 2% of total plant DNA, this sequencing depth was sufficient to achieve an average coverage of at least 10×. The high sequencing depth and quality achieved here are critical for accurate *de novo* assembly of plastomes, particularly for resolving the complex inverted repeat (IR) boundaries, which are particularly prone to assembly errors. Plastomes were assembled *de novo* using GetOrganelle v1.7.7.0 (k-mer = 85), with subsequent gap closure and polishing performed in SPAdes v3.15.5. Annotation was carried out with the Plastid Genome Annotator (PGA) (Qu et al., 2019) and manually curated in Geneious Prime v9.0.2, with special attention to IR boundaries.

### Phylogenetic analysis

The final dataset consisted of 177 accessions (175 ingroup and 2 outgroup), representing 165 species across 23 genera. These were aligned using MAFFT v7.520 (Katoh and Standley, 2013) with the L-INS-i algorithm, then concatenated and trimmed to remove regions with >30% gap frequency. ModelTest-NG v1.0.0 (Darriba et al., 2019) was used to select the optimal nucleotide substitution model under a partitioned-by-codon scheme, with the GTR+G model chosen based on AICc. Maximum likelihood (ML) analysis was conducted using RAxML-NG v1.2.0 (Kozlov et al., 2019) with 1000 rapid bootstrap replicates. Bayesian inference (BI) was performed in MrBayes v3.2.7 (Ronquist et al., 2012), running four independent Markov chains for 50 million generations and sampling every 1000th iteration. After discarding the first 25% of samples as burn-in, convergence was assessed using Tracer v1.7.3, ensuring that all effective sample size (ESS) values were ≥200 and that the potential scale reduction factor (PSRF) approximated 1.0. A coalescent-based species tree was reconstructed with ASTRAL-III v5.7.10 (Zhang et al., 2018), incorporating gene trees retaining nodes with ≥70% bootstrap support. Tree visualization and editing were performed using FigTree v1.4.4 and the online platform Chiplot (Xie et al., 2023).

### Molecular dating with TreePL

TreePL (Sanderson, 2002) was used to estimate divergence times under the penalized likelihood rate-smoothing model (Smith and O’Meara, 2012). The analysis was based on the Maximum likelihood (ML) tree inferred from complete plastome sequences. Six calibration points were applied to constrain the molecular clock, comprising four fossils: one leaf fossil and three pollen fossils (Zhang et al., 2021; Schuster et al., 2013; Manchester and O’Leary, 2010), one secondary calibration, and the stem node age of Polygonaceae, all based on previously published fossil evidence (Zhang et al., 2022a). Calibration constraints were implemented as hard bounds with minimum and maximum ages: Polygonaceae stem node (min = 94 Ma, max = 111 Ma), Polygonaceae crown node (min = 66 Ma, max = 72.1 Ma), crown node of the *Persicaria*/*Bistorta*/*Koenigia* clade (min = 77.5 Ma, max = 55.8 Ma), *Polygonum* crown node (min = 11.6 Ma, max = 5.3 Ma), *Calligonum* crown node (min = 5.3 Ma, max = 2.6 Ma), and *Muehlenbeckia* crown node (min = 22 Ma, max = 19 Ma). The smoothing parameter (λ) was optimized through cross-validation across 13 logarithmically spaced values (ranging from 10⁻^6^ to 10^6^). To assess uncertainty in node ages, we performed dating on 1000 ML bootstrap trees under a GTR+Γ model. The resulting timetrees were summarized into a maximum clade credibility tree using TreeAnnotator v2.7.2 (Bouckaert et al., 2019), with mean node ages and 95% highest posterior density (HPD) intervals annotated.

### Ecological niche modeling

We compiled species occurrence records from the Global Biodiversity Information Facility (GBIF; https://www.gbif.org), the Flora of China (http://www.iplant.cn/), and our own georeferenced field surveys. Duplicate records were removed, spatial errors (e.g., coordinates falling in oceans) were corrected, and spatial autocorrelation was reduced by applying a 10 km grid-cell thinning procedure using SDMtoolbox v2.4. Ecological niche models were constructed separately for each species and for each of three periods-the present (1970 - 2000), the Mid-Holocene (MH, ~6,000 years BP), and the Last Interglacial (LIG, ~120,000–140,000 years BP)-using MaxEnt v3.4.1. Model inputs included 19 bioclimatic variables from WorldClim v2.1 at a 2.5−arc−minute resolution, and key soil properties (dominant soil water regime and available water capacity). For each species and period, we ran 10 replicate models with 5−fold cross−validation. Models were evaluated using the area under the curve (AUC); only those with an AUC > 0.8 were retained for subsequent niche overlap analyses to ensure reliability. The resulting habitat suitability outputs were visualized in ArcGIS Pro 3.2. Using Schoener’s D index, we quantified pairwise niche overlap between lineages to assess niche dynamics and infer speciation modes, where values range from 0 (no similarity) to 1 (identical niches). An age–overlap correlation test was then performed following Fitzpatrick & Turelli (Fitzpatrick and Turelli, 2006), implemented in ENMtools. Mean niche overlap values were calculated for each node in the phylogeny, and a Monte Carlo permutation test (1000 simulations) was used to estimate the significance of the correlation between overlap and node age. Speciation modes were inferred based on the intercept and slope of the regression line fitted to overlap versus node age: an intercept > 0.5 was interpreted as evidence for sympatric speciation, whereas an intercept < 0.5 suggested allopatric or parapatric speciation; these were further distinguished by the regression slope, with parapatric speciation corresponding to a slope near or below zero and allopatric speciation to a positive slope. To enhance inference robustness, we conducted separate age–overlap analyses using both point−based and range−based niche overlap estimates.

### Evolutionary analyses

To elucidate the evolutionary trajectories underlying the adaptive radiation of Polygoneae, we reconstructed the history of key morphological and biogeographic characters using the time-calibrated plastome phylogeny. A suite of discrete functional traits - life form (herbaceous vs. woody), perianth number, inflorescence structure, fruit morphology, and woodiness (degree of lignification)-were systematically coded based on examination of herbarium specimens and a comprehensive review of taxonomic literature (**Supplementary Tables 3**, **S4**). These traits were selected for their high ecological relevance, as they are known to reflect fundamental adaptations to water stress, pollination syndromes, and dispersal mechanisms across the tribe’s diverse habitats. Ancestral states for these traits were estimated under the Markov k-state 1-parameter (Mk1) model in Mesquite v3.81 (http://www.mesquiteproject.org), with statistical uncertainty assessed via 1,000 stochastic character mappings, allowing us to identify the number and timing of key evolutionary transitions. For biogeographic inference, we applied the Statistical Dispersal-Extinction-Cladogenesis (S-DEC) model in RASP v4.2 (Yu et al., 2015). Extant species distributions were assigned to six major biogeographic regions (East Asia, North America, Europe, West Asia, Africa, Oceania, and Central Asia) based on synthesized data from GBIF, IUCN, and published floristic records.

### Phylogenetic signal and selection analyses

Phylogenetic signal for continuous morphological traits (e.g., life history, pollen morphology, life form) was assessed using Blomberg’s *K*-statistic in the R package *phytools*, with significance evaluated via 1000 permutation tests (p < 0.05), following the method of Blomberg et al (Blomberg et al., 2003). Multivariate morphological patterns were summarized using principal component analysis (PCA) on standardized traits with the prcomp function in R. Nucleotide diversity (π) and nonsynonymous-to-synonymous substitution rate ratio (Ka/Ks) were calculated for 61 plastid protein-coding genes (excluding pseudogenes and genes with <300 bp alignment length) using DnaSP v5.1. For Ka/Ks estimation, the Nei-Gojobori method was applied with the universal genetic code, and values were calculated per gene to identify signatures of selection (Ka/Ks > 1: positive selection; Ka/Ks = 1: neutral evolution; Ka/Ks < 1: purifying selection).

### Anatomical preparation and analysis

Anatomical analyses were performed on both freshly collected mature plant materials and rehydrated herbarium specimens. For fresh materials, stem segments (3–5 internodes below the apical meristem) were collected during the peak growing season and immediately fixed in FAA solution (formaldehyde: acetic acid: 70% ethanol = 5:5:90, v/v) for 48 h at 4 °C. For herbarium specimens, stem samples were rehydrated in a warm 5% aqueous glycerin solution for 24–48 h before fixation. All samples were then dehydrated through a graded ethanol series, cleared in xylene, and embedded in paraffin wax. Transverse sections (8 - 10 μm thick) were cut using a rotary microtome (Leica RM 2265), mounted on glass slides, and stained with 0.05% toluidine blue O (w/v in phosphate buffer, pH 6.8). Slides were permanently sealed with a coverslip using Permount mounting medium. High-resolution digital images were captured using a slide scanner (3D HISTECH) to ensure comprehensive and consistent imaging of the entire section. Quantitative measurements of tissue proportions and cell dimensions were performed on at least three biological replicates per species using ImageJ v1.53a (Schneider et al., 2012).

## Results

### Phylogeny of the tribe Polygoneae

**FIGURE 1:**
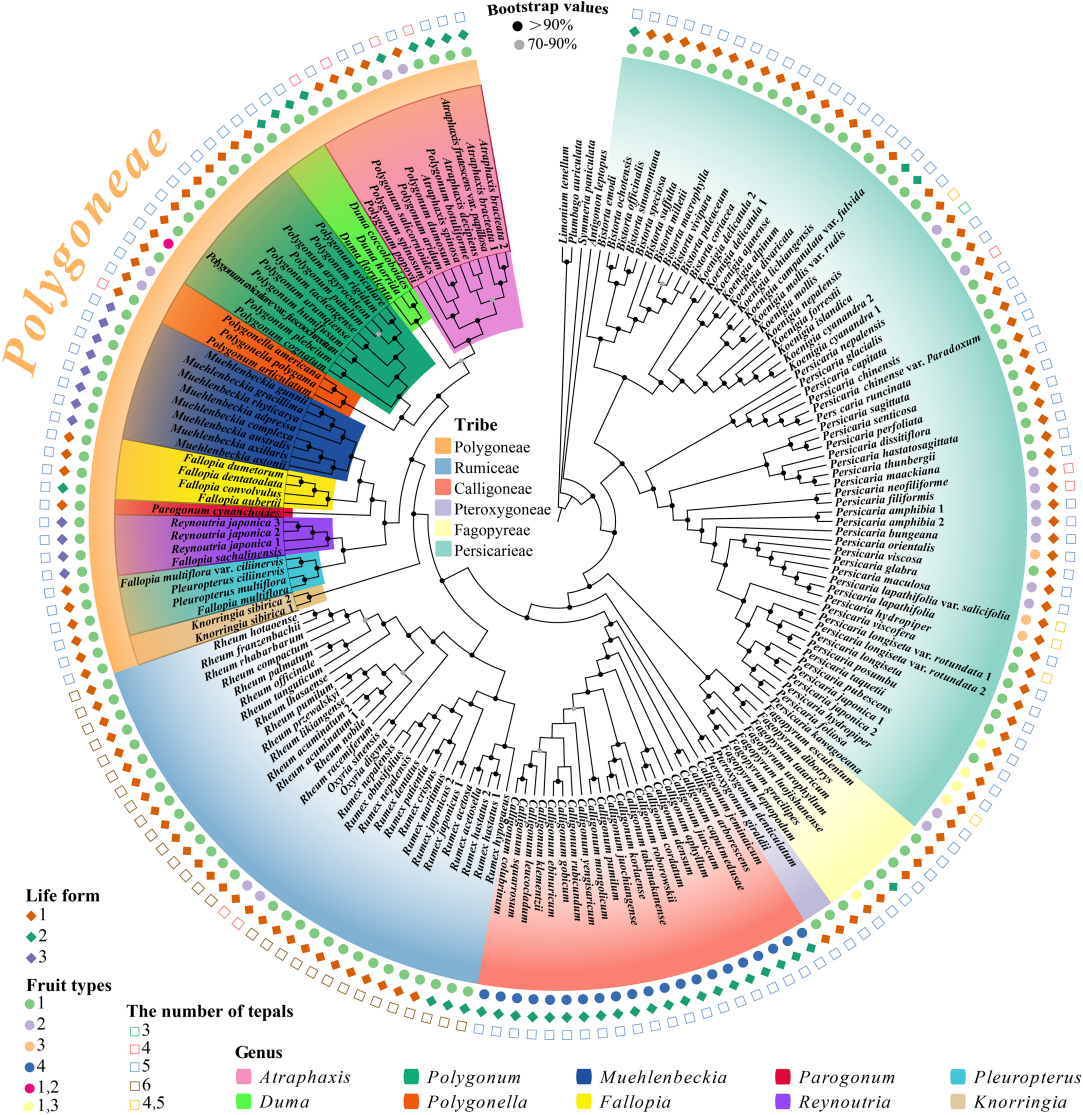
Molecular phylogenetic tree of Polygonaceae based on plastomic sequences using maximum likelihood and Bayesian inference. The tree uses *Limonium tenellum* and *Plumbago auriculata* as outgroups. Node support values are denoted by solid circles: black solid circles represent BS ≥ 90 and PP ≥ 0.95, while gray solid circles represent BS < 90 or PP < 0.95. The tree is color-coded for 6 major clades: Polygoneae, Rumiceae, Calligoneae, Pteroxygoneae, Fagopyreae, and Persicarieae. This study particularly focuses on the orange-colored branch representing Polygoneae. Within the Polygoneae clade, ten genera are recognized and distinguished by different colored blocks: *Polygonum*, *Muehlenbeckia*, *Parogonum*, *Pleuropterus*, *Atraphaxis*, *Duma*, *Polygonella*, *Fallopia*, *Reynoutria*, *and Knorringia.* Different morphological characters are denoted by distinct symbols outside the phylogenetic tree: life forms (numbered 1-3) are shown as solid-colored diamonds, where 1 represents herbaceous, 2 represents woody, and 3 represents liana; fruit types (1-4) as solid-colored circles, where 1 indicates trigonous fruits, 2 indicates biconvex fruits, 3 indicates biconcave fruits, and 4 indicates achenes with spines; and numbers of tepals (3-6) as hollow squares with different colors.

**FIGURE 2:**
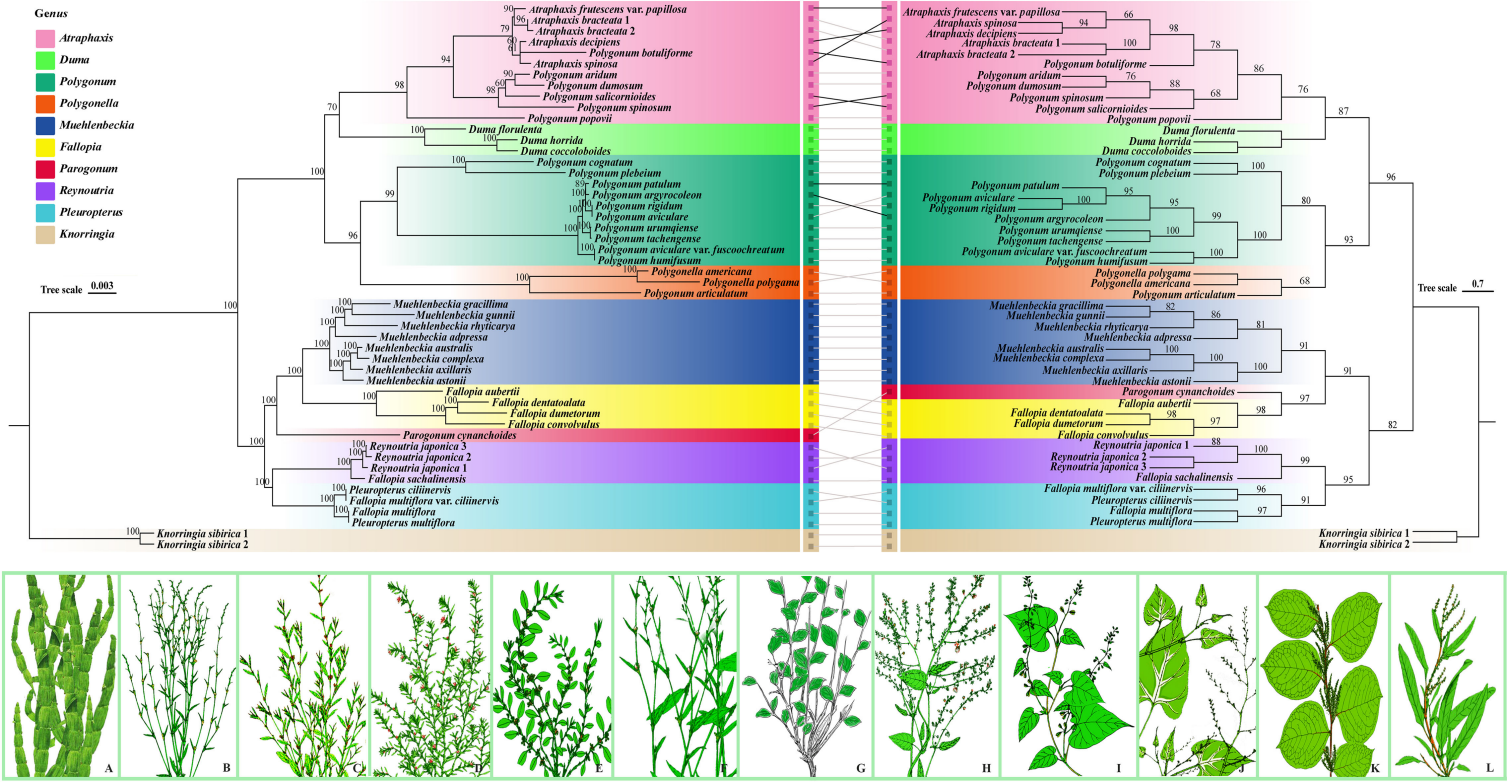
Phylogenetic relationships and morphological diversity of Polygoneae. The left panel shows the plastid genome-based maximum-likelihood (ML) phylogenetic tree, while the right panel displays the coalescent-based species tree derived from combined datasets. Line definitions: (1) Gray lines: Exclusively link corresponding species across the two phylogenies (no implication of topological conflict); (2) Black lines: Specifically highlight topological conflicts (e.g., discordant sister group relationships) between the two trees. Color-coded bars align consistent clades between the two phylogenies for intuitive comparison. The bottom panels show hand-drawn illustrations of representative species morphology: **(A)** *Muehlenbeckia platyclada*; **(B)** *Polygonum patulum*; **(C)** *Polygonum aviculare*; **(D)** *Polygonum plebeium*; **(E)** *Polygonum cognatum*; **(F)** *Polygonum argyrocoleon*; **(G)** *Atraphaxis frutescens*; **(H)** *Fallopia aubertii*; **(I)** *Fallopia cynanchoides*; **(J)** *Pleuropterus multiflorus*; **(K)** *Reynoutria japonica*; and **(L)** *Knorringia sibirica*. Numbers at nodes indicate bootstrap support values. All illustrations are copyrighted by the authors.

Phylogenetic analyses of all six tribes of Polygonoideae, conducted using both maximum likelihood (ML) and Bayesian inference (BI), resolved the internal relationships within Polygoneae with exceptionally high statistical support (**Figure 1**, **Supplementary Figures S1**, **2**). The resulting species tree is largely congruent with established taxonomic concepts while clarifying generic boundaries and revealing novel evolutionary relationships (**Figure 2**). This comprehensive plastome dataset provided robust resolution for several historically contentious nodes and elucidated the systematic positions of previously disputed or understudied lineages. Most key nodes received maximum support, confirming the reliability of the phylogenetic reconstruction. Within Polygoneae, ten highly supported subclades were identified, with *Polygonum* robustly resolved as polyphyletic. Six species (*P. botuliforme*, *P. dumosum*, *P. aridum*, *P. salicornioides*, *P.* sp*inosum*, and *P. popovii*) formed a distinct, well-nested clade within *Atraphaxis*, supporting their reclassification into this genus (**Figure 1** pink clade). However, the phylogenetic placement of several species in the *Atraphaxis* clade was discordant between the plastome tree and the coalescent-based species tree (**Figure 2**, pink clade), with conflicting nodes showing low support (BS < 70%, PP < 0.9). The combined *Duma* + *Atraphaxis* clade was recovered as sister to the *Polygonum* + *Polygonella* lineage. *Parogonum cynanchoides*, which belongs to the red-colored clade (**Figure 1**) and is characterized by intermediate traits (e.g., capitate stigma densely covered with papillae that combine features of *Muehlenbeckia* and *Fallopia*), was identified as the evolutionary link between two major radiations: one comprising *Muehlenbeckia* (blue clade) and core *Fallopia* species (yellow clade), and the other containing *Reynoutria* (purple clade) and *Pleuropterus* (light blue clade). The *Fallopia* clade included only *F. aubertii*, *F. convolvulus*, *F. dentatoalata*, and *F. dumetorum*. The species previously treated as *F. multiflorus* and *F. multiflora* var. *ciliinervia* did not cluster within *Fallopia* but rather formed a distinct clade sister to *Reynoutria*, supporting their placement in *Pleuropterus* (as *Pl. multiflorus* and *Pl. multiflora* var. *ciliinervia*). These results not only question the existing delimitation of *Fallopia* but also provide strong support for assigning *F. multiflorus* and *F. multiflora* var. *ciliinervia* to *Pleuropterus* (**Figures 1**, **2**).

### Ancestral state reconstructions and biogeographic patterns

**FIGURE 3:**
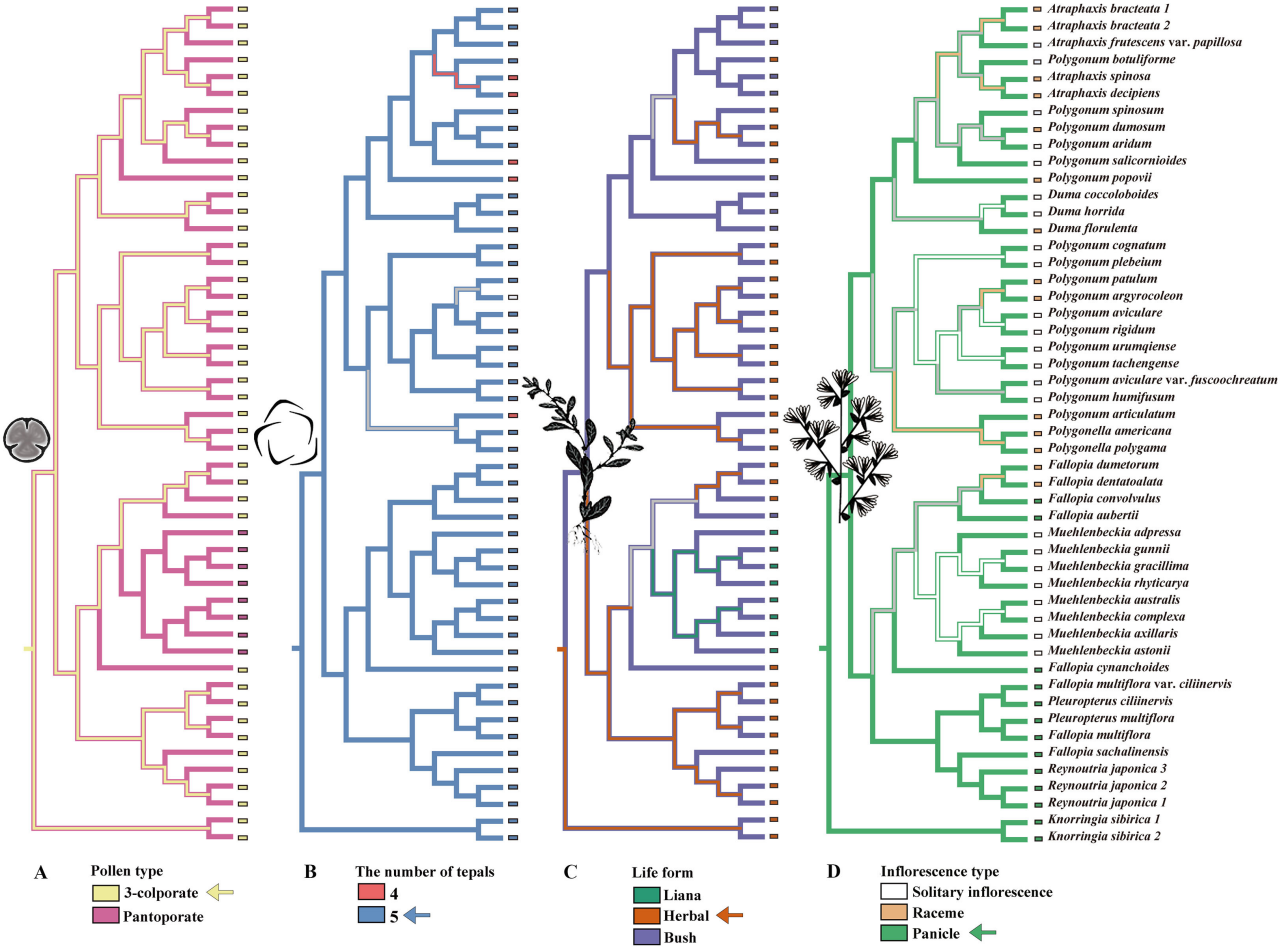
Ancestral state reconstruction of key morphological characters in Polygoneae mapped onto the plastid phylogeny. **(A)** Pollen type evolution showing transition from 3-colporate to pantoporate pollen types; **(B)** perianth number variation displaying evolutionary shifts from the ancestral state of five tepals to four tepals among different lineages; **(C)** Life form evolution demonstrating multiple independent transitions from herbaceous to woody shrub and liana growth habits, reflecting adaptations to diverse ecological niches; **(D)** Inflorescence type progression showing evolution from panicle inflorescences to solitary and racemose arrangements. All illustrations are copyrighted by the authors.

Ancestral state reconstruction within the inferred biogeographic context revealed a pronounced spatiotemporal pattern of evolution. Analyses strongly support that the ancestral Polygoneae lineage in the Paleogene was characterized by a tricolporate pollen type, an herbaceous habit, paniculate inflorescences, and a five-merous perianth (**Figure 3**). The ancestral fruit morphology, however, could not be confidently inferred, likely due to high homoplasy in fruit characters (e.g., ornamentation of achenes) across Polygoneae and limited fossil calibration for fruit traits. Phylogenetic analysis of pollen characters confirmed that the tricolporate condition, characterized by three distinct colpi each bearing a central pore, represents the ancestral state for the tribe across multiple analytical approaches. This ancestral form is highly conserved, remaining the predominant pollen type across most extant clades within Polygoneae. Within the *Atraphaxis*, the transition from five-merous to four-merous perianth represents one instance of the broader evolutionary pattern of floral organ reduction. This group exhibits both perianth types, with the four-merous condition emerging during its diversification, reflecting a convergent evolutionary trend observed across multiple lineages. Growth form reconstruction strongly supports an herbaceous habit as ancestral, with woody forms (including shrubs and lianas) evolving multiple times independently in arid and montane lineages. Similarly, paniculate inflorescences were reconstructed as the ancestral condition and are retained in most major clades. The independent shifts to solitary inflorescences occurred in at least three separate arid-adapted subclades, suggesting convergent evolution in response to dry habitats (**Figure 3**). Ancestral range reconstruction, based on a seven-region biogeographic framework for Asia (East Asia, Central Asia, North Asia, West Asia, South Asia, Southeast Asia, and the QTP), identified East Asia, particularly Southwest China, as the ancestral area for Polygoneae (**Supplementary Figure 3**). This region serves as both the distribution center and diversity center for Polygoneae. From this biogeographic cradle, multiple dispersals into Central Asia, West Asia, and the Himalayan foothills (South Asia) occurred during the Oligocene and Miocene (**Supplementary Figure 4**). These expansions were consistently associated with the repeated and independent evolution of key xeric adaptations.

### Anatomical structure and patterns of lignification

**FIGURE 4:**
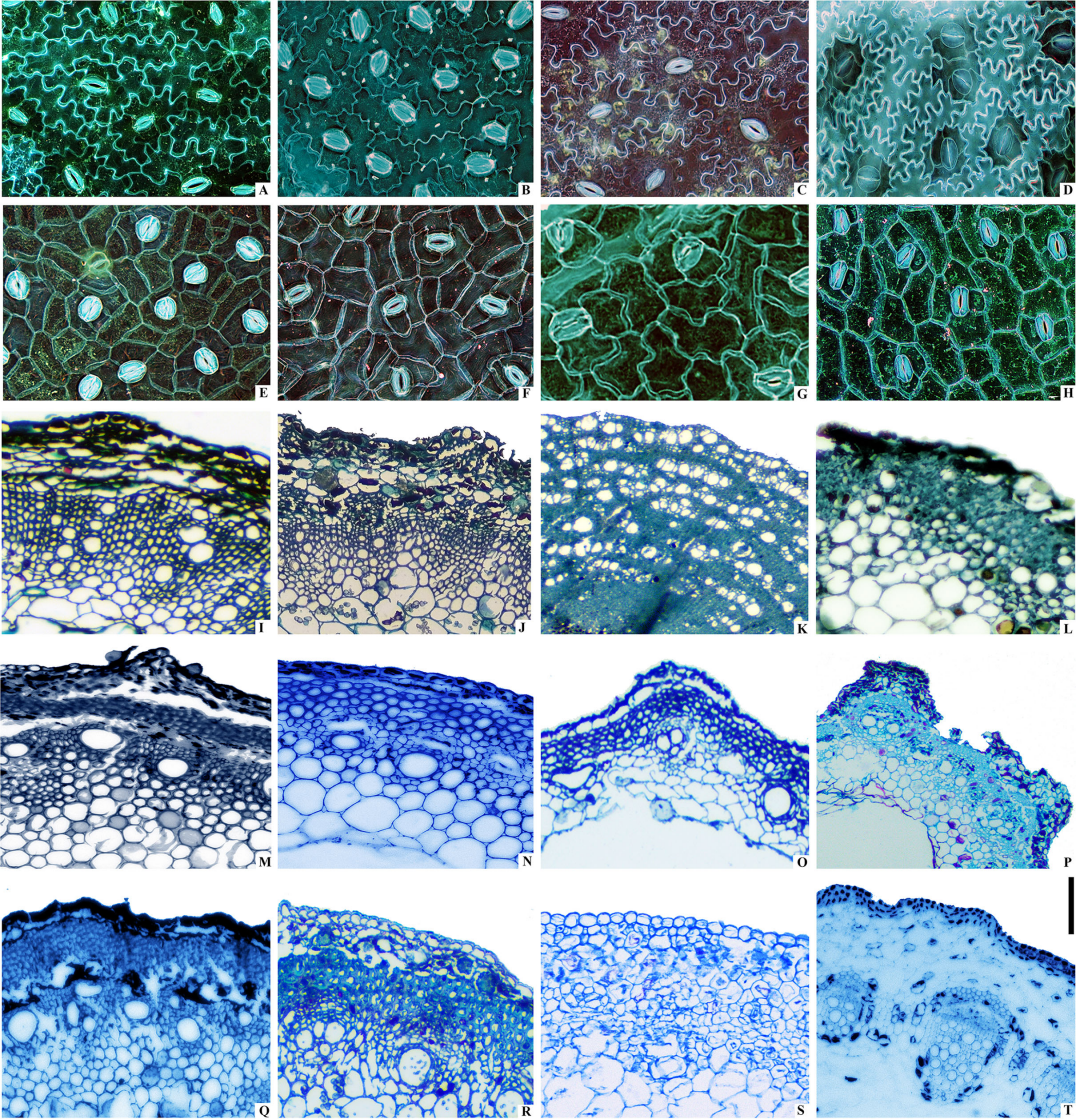
Anatomical micromorphology of leaf epicuticle and stem transverse sections in Polygoneae. Panels **(A-H)** show leaf lower epidermal micromorphology: **(A)** *Fallopia dentatoalata*; **(B)** *Fallopia convolvulus*; **(C)** *Fallopia dumetorum*; **(D)** *Reynoutria japonica*; **(E)** *Pleuropterus multiflorus*; **(F)** *Pleuropterus multiflorus* var. *ciliinervis*; **(G)** *Parogonum cynanchoides*; **(H)** *Knorringia sibirica*. The epidermal cells exhibit various arrangements. Panels **(I-T)** display stem transverse sections: **(I)** *Polygonum aviculare*; **(J)** *Polygonum humifusum*; **(K)** *Polygonum popovii*; **(L)** *Atraphaxis frutescens* var. *papillosa*; **(M)** *Fallopia aubertii*; **(N)** *Fallopia dentatoalata*; **(O)** *Fallopia dumetorum*; **(P)** *Reynoutria japonica*; **(Q)** *Pleuropterus multiflorus*; **(R)** *Pleuropterus multiflorus* var. *ciliinervis*; **(S)** *Pteroxygonum denticulatum*; **(T)** *Knorringia sibirica*. The stem sections reveal organized tissue layers. Scale bars: 20 μm for **(A-H)**, 100 μm for **(I-T)**.

The abaxial leaf epidermal micromorphology of Polygoneae revealed key traits linked to phylogeny and ecology (**Figure 4**). Epidermal cells diverged into two types: undulate cells (**Figures 4A–D, G**) with curved margins enhancing intercellular interlocking, and polygonal cells (**Figures 4E, F, H**) with straight margins. All had anomocytic stomata, but with variations: large, sparse stomata in B, E, G, H; small, dense ones in A, F. Phylogenetically, polygonal cells (**Figures 4E, F, H**) suggest ancestral traits, while undulate cells (**Figures 4A–D, G**) reflect a derived, herbaceous-adapted trend aiding clade inference. Anatomical examination of stems across Polygoneae revealed significant variation in lignification patterns, closely corresponding to life form and ecological adaptation. In the annual herb *Polygonum aviculare* (**Figure 4I**), lignification was minimal and restricted primarily to the vascular bundles and adjacent sclerenchyma, collectively comprising only 15 - 20% of the stem’s cross-sectional area. The vascular bundles featured solitary vessels and slender fibers (8 - 12 μm in diameter). In stark contrast, the perennial shrub *A. frutescens* var. *papillosa* displayed a prominent lignification core, with lignified tissues accounting for 35 - 40% of the stem’s cross-sectional area (**Figure 4L**). A continuous cambium has produced abundant secondary xylem, visible as concentric growth rings. The xylem features dense vessels (20 - 30 μm in diameter), which are clearly discernible within the rings. These anatomical traits are consistent with a woody shrub adapted to arid environments. The semi-shrubby liana *F. aubertii* displayed a uniform, miniaturized lignification pattern, with lignified tissues occupying 25 - 30% of the stem area (**Figure 4M**), which is intermediate between the herbaceous *F. dumetorum* (<15%) and the woody *Atraphaxis* (35 - 40%). Conversely, *F. dentatoalata* exhibited significantly enlarged vascular cells (**Figure 4N**), with vessel diameters (35 - 45 μm) and tracheid lengths more than double those of *F. aubertii*, suggesting an adaptation for enhanced hydraulic efficiency in moist, forest-edge habitats. The herbaceous *F. dumetorum* retained ancestral anatomical features. Lignification was confined to bundle-sheath fibers and sparse vessels (5–8 per mm²), which collectively accounted for less than 15% of the stem cross-sectional area. Additionally, this species had a vast, starch-rich pith (40 - 50% of stem area) (**Figure 4O**). In contrast, the robust perennial herb *Reynoutria japonica* exhibited a stem anatomy dominated by mechanical reinforcement. The vascular bundles, concentrated around the pith (a perimedullary pattern), were heavily lignified. They featured radially arranged vessels and were embedded within a massive cylinder of thick-walled fibers (20 - 25 μm in diameter). Collectively, these lignified tissues constituted an estimated 60 - 70% of the stem cross-sectional area (**Figure 4P**). This extraordinary investment in supporting tissue underpins the stiff, hollow stems characteristic of this fast-growing invasive species. A bimodal lignification pattern was observed in *Pl. multiflora* and its variety *Pl. multiflora* var. *ciliinerve*, featuring an outer ring of large-diameter fibers (30 -45 μm) and scattered small lignified cells in the central vascular cylinder (**Figures 4Q, R**). *Pteroxygonum giraldii* displayed a strictly centralized pattern, with a highly lignified core (60 - 70% of the area, lignin content 25 - 30%) and non-lignified peripheral tissues (**Figure 4S**). Finally, the halophyte *Knorringia sibirica* exhibited a fasciculate-ring structure, with 8–10 discrete lignified vascular bundles arranged in a ring, separated by large parenchyma cells containing crystal clusters, likely related to ion balance in saline environments (**Figure 4T**).

### Ecological niche modeling

**FIGURE 5:**
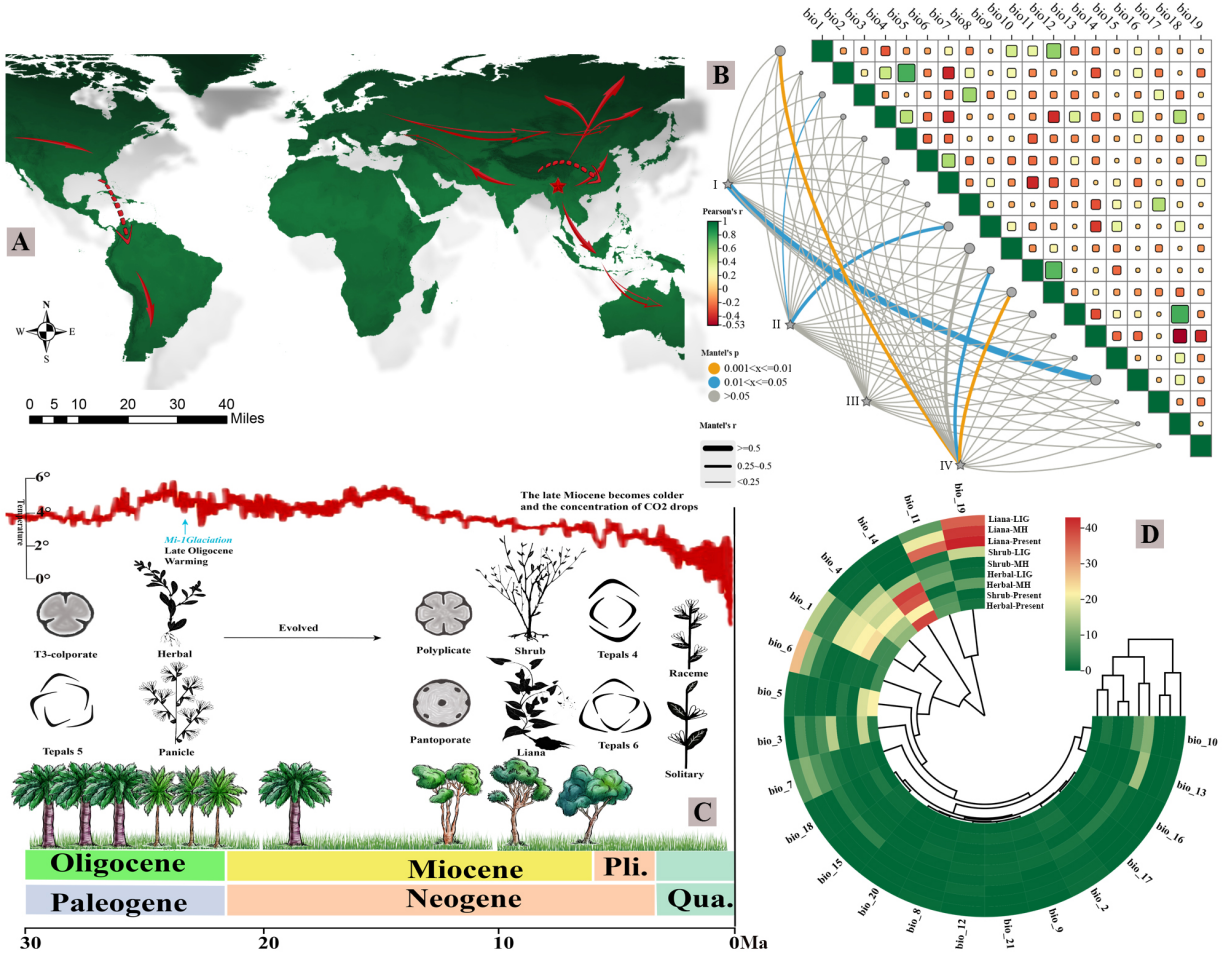
Integrated phylogeographic and ecological niche modeling of Polygoneae diversification across temporal scales. **(A)** Global dispersal trajectories showing migration routes of different life forms during the Last Interglacial (LIG), Mid-Holocene (MH), and Present. Hollow arrows represent woody shrubs, solid arrows indicate herbaceous species, and dashed arrows show liana dispersal patterns. **(B)** Ecological niche modeling visualization with PCA analysis. Panel I shows morphological trait variation, Panel II displays distribution latitude/longitude coordinates, Panel III represents life cycle traits, and Panel IV illustrates chloroplast genome component sizes across taxa. **(C)** Ancestral trait reconstruction integrated with Paleogene to Quaternary climate change, showing CO_2_ concentration (red curve) and temperature fluctuations (black curve). Morphological transitions include: pollen evolution from 3-colporate to polyporate and pantoporate types; tepal number reduction from the ancestral five-tepal condition to derived four- or six-tepal forms; life form shifts from herbaceous to shrub and liana habits; and inflorescence diversification from paniculate to solitary and racemose arrangements. **(D)** Circular phylogenetic tree with trait evolution heatmap (scale: –40 to +40), illustrating the correlation between phylogenetic relationships and adaptive trait values.

Ecological niche modeling (ENM) using MaxEnt revealed significant niche differentiation among the studied genera of Polygoneae, driven by distinct bioclimatic variables (**Figure 5**; **Supplementary Tables 5**, **6**). All models showed high predictive accuracy (AUC > 0.88, **Supplementary Table 7**). Variable importance analysis identified primary climatic constraints for each genus, revealing a spectrum of adaptive strategies from extreme aridity tolerance to precipitation dependency. Arid-adapted shrubs, such as *Atraphaxis* and *Polygonella*, were principally limited by temperature extremes. *Atraphaxis* niche was most influenced by the maximum temperature of the warmest month (bio5, 33.3%) and the mean temperature of the coldest quarter (bio11, 17.2%), consistent with its distribution in continental deserts. *Polygonella* exhibited an even stronger specialization to aridity, with its distribution overwhelmingly constrained by precipitation of the driest month (bio14, 51.0%). In contrast, genera from mesic habitats showed stronger dependencies on precipitation regimes. The invasive herb *Reynoutria* was predominantly limited by precipitation of the driest month (bio14, 67.4%), were reliably projected by ENM models (AUC > 0.88), underscoring a critical drought tolerance threshold (**Supplementary Table 7**). The liana *Fallopia* was highly sensitive to precipitation during the wettest periods (bio6, 38.9%; bio12, 21.3%). The temperate genus *Muehlenbeckia* was primarily constrained by the minimum temperature of the coldest month (bio6, 43.9%), reflecting its adaptation to cooler climates. These climatic niches corresponded to distinct biogeographic patterns. *Atraphaxis* showed the highest suitability in the arid and semi-arid regions of Central Asia (e.g., Iranian Plateau, Tianshan Mountains). *Duma*, whose niche was defined by isothermally (bio3, 52.4%) and dry-season precipitation (bio14, 19.7%), was most suitable in the climatically seasonal and fire-prone ecosystems of southeastern Australia. The *Muehlenbeckia* displayed broad plasticity, occupying distinct niches in South America, Oceania, and East Asia, linked by a shared tolerance to cold minima. Projections to past climates (Last Interglacial and Mid-Holocene) revealed differing evolutionary trajectories among life forms. Herbaceous lineages exhibited notable niche conservatism, with precipitation of the driest month (bio14) remaining the dominant constraint across all periods (39.9 - 39.4%). The woody species underwent a fundamental niche shift, transitioning from temperature-limited distributions in past climates to stronger precipitation-limited distributions under current conditions. Lianas exhibited intermediate plasticity, maintaining ancestral temperature sensitivities while evolving new dependencies on contemporary precipitation regimes.

### Polygoneae phylogenetic signal and adaptive evolution

Phylogenetic signal analysis of eight morphological characters revealed distinct evolutionary patterns between reproductive and vegetative traits. Blomberg’s K statistic demonstrated strong phylogenetic conservatism (K > 1, p < 0.01) in life history (annual/perennial: K = 1.69), life form (herbaceous/woody/liana: K = 1.11), and pollen morphology (pollen: K = 1.47). Inflorescence structure (K = 0.29), perianth merosity (K = 0.23), and fruit morphology (K = 0.17) showed significantly weak phylogenetic signals (p < 0.05), indicating high evolvability and widespread convergent evolution. Phylogenetic principal component analysis further elucidated the multidimensional landscape of trait evolution (**Supplementary Table 8**). The first axis (PC1) explained 86.8% of total variance and was loaded exclusively by life form, confirming that repeated evolution of woodiness represents the primary driver of morphological diversification. The second axis (PC2, 6.4% variance) was defined solely by pollen morphology, reinforcing its role as a deeply conserved phylogenetic marker. Subsequent axes (PC3 and PC4) captured finer-scale ecological differentiation through synergistic shifts among labile traits, including life history, fruit morphology, and perianth characteristics, reflecting niche partitioning beyond the fundamental woodiness-aridity adaptation (**Supplementary Table 9**). The photosystem I gene *psaI* (Ka/Ks = 1.40) showed strong signatures of positive selection, potentially reflecting adaptation to maintain photosynthetic efficiency under water stress. Additionally, *accD* (Ka/Ks > 0.7) and *ycf2* (Ka/Ks > 0.6) indicated adaptive evolution in lipid metabolism and plastid translation machinery, respectively (**Supplementary Table 10**).

### Spatiotemporal dynamics of habitat suitability

**FIGURE 6:**
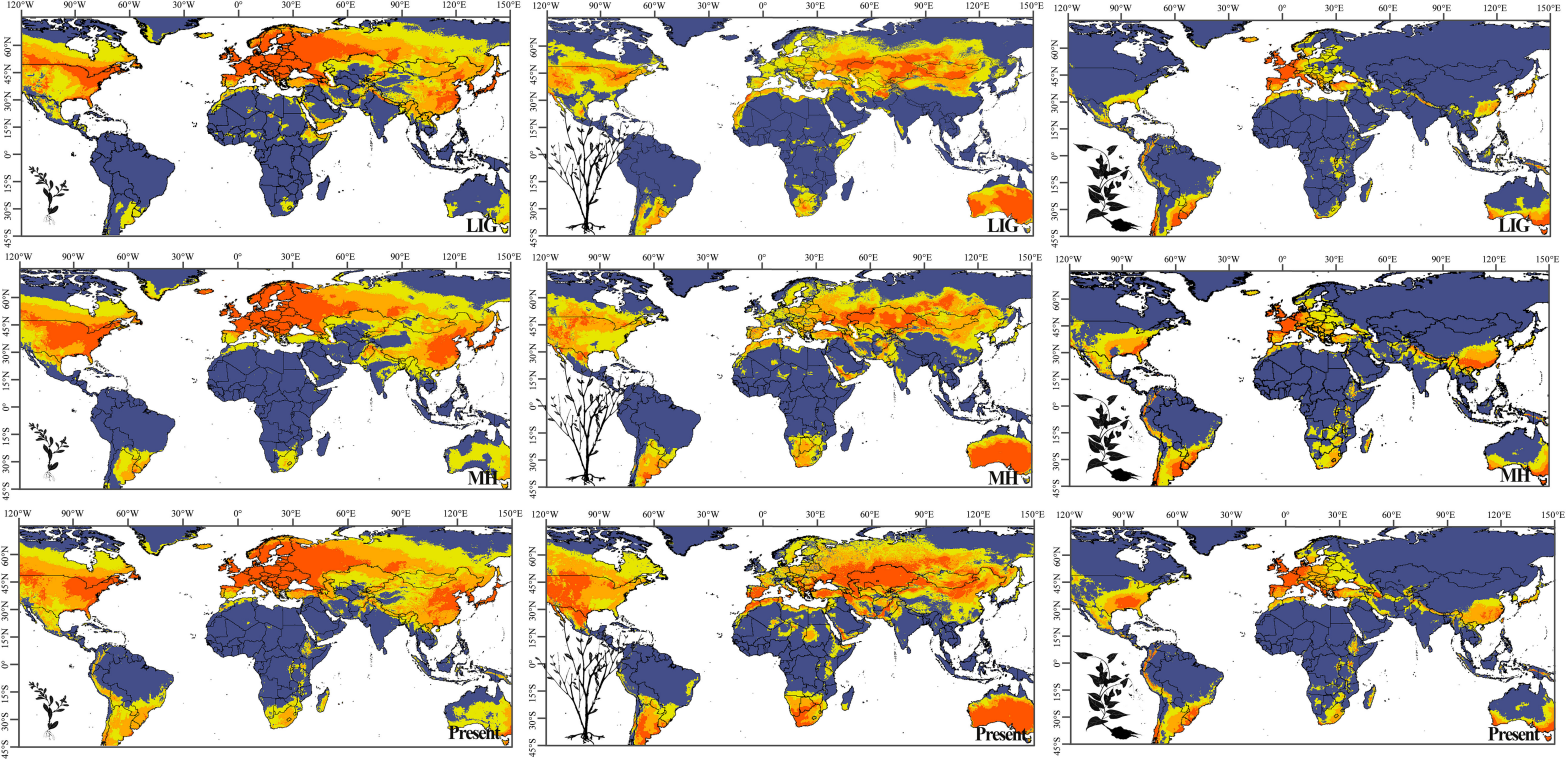
Ecological niche modeling results showing habitat suitability distribution for three life forms of Polygoneae across temporal periods. The map displays four suitability categories: highly suitable (red), moderately suitable (orange), less suitable (yellow), and non-suitable (dark blue).Nine-panel global distribution maps showing ecological niche modeling for three Polygoneae life forms across three time periods. Rows represent time periods (Last Interglacial, Mid-Holocene, Present); columns represent life forms (herbaceous, woody shrub, liana).

Ecological niche models were constructed for each genus using MaxEnt v3.3.3k with uncorrelated bioclimatic variables, and all models yielded an area under the curve (AUC) score > 0.80, confirming sufficient reliability for subsequent niche overlap analyses (**Supplementary Table 7**). Ecological niche models reconstructed across the Last Interglacial (LIG), Mid-Holocene (MH), and present day revealed striking spatiotemporal shifts in habitat suitability that varied markedly among life forms (**Figure 6**). During the warm LIG period, all three life forms exhibited broadly suitable conditions across mid-to-high latitudes of the Northern Hemisphere, with herbaceous, woody, and liana taxa sharing largely overlapping distributions characterized by contiguous zones of high suitability spanning Eurasia and North America. The pattern suggests that the climatic optimum during the LIG supported diverse life forms across extensive geographic ranges with minimal niche differentiation. The transition to the MH, however, brought pronounced southward contraction and fragmentation of suitable habitats, with the magnitude of these shifts differing substantially among life forms. Woody and liana forms experienced the most dramatic range reductions, with their core suitable areas retreating into isolated refugia concentrated in Central and Eastern North America, Southern and Central Europe, and East Asia, resulting in a 45 - 60% reduction in highly suitable habitat area compared to the LIG. In marked contrast, herbaceous forms demonstrated greater resilience to these climatic changes, maintaining relatively continuous distributions with only modest range contractions (approximately 25 - 30% reduction). Herbaceous taxa retained considerable habitat suitability not only in Northern Hemisphere refugia but also expanded or maintained presence in Southern Hemisphere regions, including Africa and Australia, suggesting their capacity to track suitable conditions across broader latitudinal gradients. Under current climatic conditions, these divergent trajectories have intensified further. Woody and liana forms now occupy consolidated ranges within specific mid-latitude zones (primarily 30 - 50°N), exhibiting increased fragmentation with suitable habitat patches becoming smaller and more isolated—a pattern likely reflecting the combined effects of modern climate constraints, particularly reduced precipitation in continental interiors, and intensifying anthropogenic pressures including habitat conversion and land use change. Herbaceous forms, conversely, continue to exhibit the broadest ecological amplitude, with highly suitable habitats spanning multiple continents and extending across a wide latitudinal range (20 - 60°N and into southern temperate zones). The pattern aligns with their documented niche conservatism, superior dispersal capabilities mediated by smaller propagule size and wind dispersal mechanisms, and greater physiological tolerance to environmental variability, collectively enabling them to track suitable conditions across broader geographic and climatic gradients than their woody and liana counterparts.

## Discussion

### Phylogenetic relationships within Polygoneae

Our phylogenomic framework robustly resolves long-standing taxonomic disputes within Polygoneae, corroborating earlier molecular assessments that revealed extensive polyphyly of *Polygonum* (Schuster et al., 2015; Zhang et al., 2022c, Zhang et al., 2022b). The robust support for clade pink indicates the reclassification of secondarily lignified species (e.g., *P. botuliforme*, *P. dumosum*, *P. aridum*, *P. salicornioides*, *P.* sp*inosum*, and *P. popovii*) into *Atraphaxis*. Their nested position within *Atraphaxis* is corroborated by key derived morphological synapomorphies, notably sessile or short-petiolate linear leaves, slightly revolute margin, and bilobed stipular sheaths (Yurtseva et al., 2017, Yurtseva et al., 2022). Anatomically, these reclassified taxa (e.g., *P. popovii*) exhibit a xylem-dominated syndrome, with substantial secondary lignification (up to 40%, **Figures 4K, L**) and narrow vessel diameters (15 - 20 μm). The substantial secondary lignification likely provides additional mechanical reinforcement against xylem implosion under extreme water stress in the arid Tianshan corridor (Cabon et al., 2020; Lloret et al., 2012). These traits contrast sharply with herbaceous *Polygonum* s.s. (e.g., *P. aviculare*), which exhibits limited lignification (15 - 20% of stem, **Figure 4I**), confirming the artificiality of their traditional placement in *Polygonum* (**Figures 1**, **4**). Our phylogenetic analysis reveals significant incongruence between current subgeneric classifications and evolutionary relationships within *Atraphaxis*. The traditional morphology-based groupings, primarily distinguished by perianth number (four versus five), exhibit low phylogenetic signal (K = 0.23), indicating substantial environmental convergence (**Figure 2**, pink clade) (Li et al., 2003). These diagnostic markers are therefore unreliable for delimiting natural lineages, resulting in paraphyletic assemblages that require comprehensive taxonomic revision to distinguish homologous traits from aridity-driven convergent adaptations (Zhang et al., 2014; Tavakkoli et al., 2014; Yurtseva et al., 2022). Although our phylogenetic analysis suggests that *Duma* may form a monophyletic group, this relationship receives only moderate statistical support. The genus is characterized by several distinctive morphological features, including spinescent branch tips and sessile, linear leaves, which have been proposed as potential synapomorphic traits (Decraene et al., 2008; Hong et al., 2005; Desjardins et al., 2023). The expanded taxon sampling, incorporating key endemic species from Xinjiang (e.g., *P. urumqiense*, *P. tachengense*) and previously unsampled taxa (e.g., *P. patulum*, *P. argyrocoleon*, *Polygonella americana*), has substantially improved phylogenetic resolution. These results provide strong support for the placement of these species and necessitate a taxonomic realignment, including new combinations such as the transfer of *Polygonella americana* to *Polygonum americanum* (Schuster et al., 2011a; Hong et al., 2005).

Within the RMF clade, phylogenetic analyses revealed deep polyphyly in *Fallopia*, necessitating substantial taxonomic revisions. The exclusion of *F. giraldii* (now *Pteroxygonum giraldii*) and the identification of *F. cynanchoides* (now *Pteroxygonum giraldii*) as a distinct transitional lineage prompted a reassessment of generic boundaries (Cao et al., 2023; Schuster et al., 2011b; Desjardins et al., 2023). The distinct generic status of *Parogonum* is robustly supported by a unique combination of morphological (stems lacking ridges but bearing dense brown indumentum, free styles, reticulate pollen) and chemical (quercetin derivatives) characters, thereby justifying its resurrection as a monotypic genus. Our data support transferring *Fallopia multiflora* and *F. ciliinervia to Pleuropterus* based on shared synapomorphies including paniculate inflorescences, brush-shaped stigmas, and reticulate pollen ornamentation (Schuster et al., 2011b, Schuster et al., 2015; Zhang et al., 2022c). These species are further distinguished from core *Fallopia* s.s. (e.g., *F. convolvulus*, *F. dumetorum*) by the absence of post-anthesis perianth enlargement and flavonoid profiles congruent with *Reynoutria*—potentially reflecting specialized metabolic adaptations to disturbed habitats (Macedo et al., 2021). To further elucidate the evolutionary patterns underlying these taxonomic rearrangements, we examined the phylogenetic signal strength across various morphological characters. The robust phylogenetic signal observed in pollen ornamentation (K = 1.47) confirms its utility as a conservative synapomorphy, in contrast to the labile vegetative traits, with reticulate patterns retained in 92% of *Fallopia* s.s. and 88% of *Reynoutria* species (Wang and Chen, 2020a). Similarly, the annual or perennial life history strategy exhibits the highest phylogenetic signal (K = 1.69, p < 0.001), reflecting pronounced niche conservatism in reproductive timing. The signal for life form was also significant but lower (K = 1.11, p < 0.05). By contrast, specific floral traits showed markedly weaker phylogenetic signals: perianth merosity (K = 0.23) and inflorescence structure (K = 0.29) both displayed significant but low values (p < 0.05), a pattern consistent with pervasive ecological convergence in floral morphology. These findings indicate that while the timing of reproduction (annual vs. perennial) is deeply conserved as a core life-history program, growth form is more evolutionarily labile and can readily converge under similar environmental pressures (Adler et al., 2014; Matthew Ogburn and Edwards, 2015). Such marked divergence in phylogenetic signal strength necessitates a critical reevaluation of morphological character weighting in Polygoneae systematics: characters exhibiting high homoplasy, including woodiness and perianth reduction, should be deemphasized in taxonomic delimitation, whereas pollen ornamentation patterns provide more robust synapomorphic evidence for defining generic boundaries (Wang et al., 2015; Blomberg et al., 2003). Pollen morphological evidence supports the taxonomic reassignment of *P. popovii*: pollen analysis reveals that *P. popovii* exhibits morphological affinities with *Atraphaxis* in grain size, aperture number, and exine ornamentation, while differing markedly from Polygonum by possessing larger pollen grains and more numerous apertures (Hong et al., 2005; Yang et al., 2020; Zhou et al., 1999). The evolutionary lability of vegetative traits has profound implications for understanding invasiveness within the RMF clade. Independent evolutionary lineages, represented by the invasive taxa *Fallopia japonica* and *Reynoutria* species, have convergently evolved invasive traits, including rapid vegetative growth and extensive rhizome systems (Schuster et al., 2011b). Convergent evolution in these taxa appears to reflect adaptive responses to comparable anthropogenic disturbances, particularly horticultural introduction and nutrient-enriched habitats (Desjardins et al., 2023; Franks et al., 2007). The perimedullary concentrated lignification pattern observed in *R. japonica* (**Figure 4P**), which combines herbaceous flexibility with woody-like mechanical strength, may contribute to its exceptional competitive ability by enabling rapid vertical growth while maintaining structural integrity under high wind loads (Mahmoudi et al., 2020; Yang et al., 2020). Understanding the phylogenetic distribution of such invasiveness-related traits can inform risk assessment protocols for horticultural introductions and guide the selection of biological control agents (Schuster et al., 2015; Verloove et al., 2021).

Phylogenetic discordance was observed among several species within the *Atraphaxis* clade (e.g., *A.* sp*inosa*, *A. botuliforme*) between the plastome and coalescent-based species trees (**Figure 2**, pink clade), with conflicting nodes showing low support (BS < 70%, PP < 0.9). Three non-mutually exclusive mechanisms may explain this discordance: first, incomplete lineage sorting (ILS), as the late Miocene–Pliocene diversification of *Atraphaxis* (10–3 Ma, **Supplementary Figure 4**) coincided with rapid Tianshan uplift and intense aridification (Barbolini et al., 2020), which compressed speciation intervals and increased the likelihood of retained ancestral polymorphisms (Zhang et al., 2022b). Second, hybridization or introgression is plausible, given that these arid-adapted species often occupy overlapping montane microhabitats in Central Asia (Xu et al., 2016), creating opportunities for interspecific gene flow. In contrast to the nuclear genome signals captured in coalescent trees, the maternally inherited plastid genomes in most angiosperms may retain a different set of signals reflecting historical hybridization (Schuster et al., 2015). Third, differential selection on plastid vs. nuclear genomes in arid environments could drive topological conflicts: plastid genes such as *psaI* (Ka/Ks = 1.40) under positive selection for drought adaptation (**Supplementary Table 10**) may exhibit accelerated evolutionary rates, altering phylogenetic placement relative to nuclear genes governing stem lignification (Xie et al., 2018). Regarding the historically problematic placement of *Fallopia cynanchoides* (now *Parogonum cynanchoides*), its unstable position reflects genuine evolutionary complexity rather than methodological artifacts (Schuster et al., 2011b; Desjardins et al., 2023). Long-branch attraction is unlikely, as our plastome tree shows no extreme branch lengths for this species (**Figure 2**). Instead, introgression is a strong candidate: *F. cynanchoides* occupies ecotonal habitats (mesic forest edges adjacent to arid slopes) along the Hengduan-Qinling corridor, a zone known for high interspecific gene flow (Tang et al., 2018), where plastid capture from *Atraphaxis* (arid-adapted) or other Polygoneae lineages could explain its variable placement (Gaynor et al., 2020). Limited informative sites in past studies (e.g., single plastid fragments) exacerbated this instability. The persistence of ambiguity even in our whole-plastome analysis suggests that the species may represent a relictual lineage with conflicting genomic signals stemming from ancient divergence. Resolving these discordances will require integrating nuclear genomic data (e.g., target enrichment or whole-genome sequencing) to disentangle ILS, hybridization, and selection, as nuclear markers (with biparental inheritance) can complement plastid data in reconstructing reticulate evolutionary histories (Zhang et al., 2021). Future studies should also include population-level sampling of *Atraphaxis* and *Pteroxygonum giraldii* to detect signatures of introgression and clarify the role of ecological adaptation in shaping genomic discordance. These results demonstrate that niche overlap was higher among closely related taxa at younger phylogenetic nodes and progressively reduced among distantly related lineages at older nodes, supporting parapatric speciation as the dominant speciation mode within Polygoneae. Consistent high niche overlap (Schoener’s D > 0.6) was observed between sister genera or closely related sympatric lineages, including *Polygonum* and *Polygonella* (D = 0.72) and *Reynoutria* and *Fallopia* (D = 0.65). In contrast, low niche overlap (D < 0.3) characterized genera pairs with extreme habitat differentiation or intercontinental geographic isolation, such as *Atraphaxis* and *Pleuropterus* (D = 0.18) and *Muehlenbeckia* and *Parogonum* (D = 0.25). Notably, *Knorringia* exhibited a distinct isolated niche, with Schoener’s D values < 0.4 relative to all other genera, a pattern reflective of its unique climatic adaptation (**Supplementary Table 11**).

### Adaptive radiation and ecological divergence in Polygoneae

The evolutionary history of Polygoneae exemplifies adaptive radiation driven by the interplay of paleoclimatic oscillations, biogeographic isolation, and trait innovation across multiple organizational levels. Molecular clock analyses date the divergence between the arid-adapted (ADP) and mesic-adapted (RMF) clades to the late Paleogene/early Neogene (**Supplementary Figures 3**, **4**). The timing of this divergence links their ecological niche partitioning to major paleoclimatic and/or orogenic events, specifically the uplift of the Qinghai-Tibet Plateau (QTP) and the intensification of the Asian monsoon (Lyu et al., 2024; Sun et al., 2014). The initial diversification of the Polygoneae coincided with the Paleocene–Eocene Thermal Maximum around 53 million years ago. Subsequent diversification was further accelerated during the early Miocene uplift of the QTP (23–16 Ma), a period of intense tectonism that created topographic heterogeneities, fragmented ancestral ranges, and generated novel arid habitats (Wu et al., 2022; Zhang et al., 2022b). The geological activity aligns with the first appearance of xerophytic traits (e.g., revolute leaf margins) in Polygoneae fossils from the Qaidam Basin (Schuster et al., 2015; Barbolini et al., 2020). The most pronounced radiation occurred during the mid-to-late Miocene (15–5 Ma), coinciding with global cooling, intensified QTP uplift, and widespread aridification across temperate regions (Cao et al., 2022; Mao et al., 2021; Cao et al., 2025). Our integrated analysis combining paleo- and contemporary ecological niche modeling with stem anatomical data indicates that this ecological divergence is structurally supported by distinct xylological syndromes (**Figure 4**). During this phase, the radiation was likely triggered by global cooling trends and intensified uplift of the QTP, which collectively drove widespread aridification across temperate regions. The Miocene pulse established a bifurcated evolutionary trajectory that persists into the present: herbaceous lineages exhibit strong niche conservatism (retention of ancestral climatic tolerances), whereas woody shrubs display dynamic niche evolution (Wu et al., 2022; Cabon et al., 2020). Consistent with this pattern, herbaceous *Polygonum* taxa exhibit broad temperature-precipitation ranges, while woody clades (e.g., *Atraphaxis*) have narrowed into aridity-specialized niches (Zhang et al., 2021; Li and Wang, 2020). Herbaceous lineages exhibit a conserved pith-dominated syndrome, characterized by large pith cavities, a thin cortex, and discrete vascular bundles with minimal secondary growth (**Figure 4**). This anatomical configuration provides multiple functional advantages: water storage capacity through parenchymatous tissues (Mauseth, 2006; Lens et al., 2022), efficient mechanical support via turgor pressure, and enhanced reproductive allocation. The significant correlation with bio14 (precipitation of the driest month; contribution rate: 37.6 - 39.9%, as determined by MaxEnt variable importance analysis) reveals that medullary water storage structures are an adaptive response to a critical ecological constraint - water limitation during the dry season (Harrison et al., 2020; Compagnoni et al., 2021). By internally buffering water deficits, this anatomy directly mitigates the primary climatic constraint on herbaceous survival. This functional trait explains its dominant role in shaping the modeled niche. This pith-based water storage enables herbaceous taxa to track specific hydrological regimes across continents. Their structural conservatism, coupled with high dispersal capacity, has maintained widespread (Zanne and Falster, 2010), continuous distributions across the Northern Hemisphere temperate belt since the Last Interglacial (LIG, ~120 ka). This persistence reflects their ability to survive Quaternary glaciations in refugia and rapidly recolonize during interglacials (Stewart et al., 2009). The Last Interglacial (LIG) was characterized by global temperatures 0.5 - 1.5 °C above pre-industrial levels, with Arctic regions experiencing 4 - 8 °C warming (Fontes et al., 2022). During this period, herbaceous Polygoneae likely expanded northward, tracking favorable moisture regimes. The consistent retention of this syndrome across phylogenetically distant herbaceous lineages underscores strong functional constraints on stem architecture in seasonally dry temperate environments (Yang et al., 2020; Pandey, 2021).

Woody shrubs (e.g., *Atraphaxis*) exhibit a contrasting xylem-dominated anatomical syndrome defined by extensive secondary growth (8–20 layers of secondary xylem), reduced pith (10 - 20% of stem diameter), and well-developed periderm. This structural reorganization represents adaptations to chronic water scarcity and thermal extremes in Central Asian deserts (**Figure 4**). Traits such as full-stem lignification (35 - 40% of stem cross-section occupied by secondary xylem) and narrow vessel diameters (15 - 20 μm) may contribute to enhanced mechanical support and reduced vulnerability to freeze-thaw embolism (Pandey, 2021). The extensive secondary growth likely provides mechanical reinforcement against xylem implosion under extreme water stress and wind loading (Pratt et al., 2007). The diversification of lignification strategies in *Atraphaxis* is tightly linked to Central Asia’s geological and climatic history (Fontes et al., 2022; Cabon et al., 2020). Molecular clock analyses indicate the most recent common ancestor (MRCA) of *Atraphaxis* emerged in the montane regions of the Dzungarian Basin and Pamir-Tianshan-Alatau mountain chain during the late Miocene (10–6 Ma). This timeframe coincides with the rapid uplift and exhumation of the Tianshan Mountains during the late Miocene (Barbolini et al., 2020), which generated marked topographic heterogeneity across the region. The resulting mosaic of slopes, aspects, and elevations likely created diverse microclimates and edaphic conditions, driving the evolution of distinct lignification strategies within the genus. This tectonic activity also reorganized the Central Asian climate, establishing distinct seasonal precipitation patterns and further aridifying inland China (Wu et al., 2022; Miao et al., 2022). Hyper-arid desert specialists (e.g., *A. popovii*) evolved whole-stem lignification, characterized by a high proportion of secondary xylem (**Figure 4K**). This extensive lignification confers critical biophysical resilience by providing the mechanical strength necessary to withstand extreme negative pressure and maintain hydraulic integrity under severe, sustained drought (Pratt et al., 2007). Semi-arid grassland endemics (e.g., *A. bracteata*) evolved concentrated secondary xylem to balance mechanical support and hydraulic efficiency (Xu et al., 2016). Climbing species occupy an intermediate niche, integrating moderate pith development (30 - 50% of stem diameter) with plastic secondary growth (1–14 cell layers). This plasticity enables colonization of mesic forest-edge habitats along the Quaternary refugial corridor of the Hengduan-Qinling-Yanshan mountains. The redefined genus *Pleuropterus* (e.g., *P. multiflorus*) exhibits a mixed lignification pattern: large lignified cells in the outer cortex and smaller lignified cells in the central vascular cylinder, distinguishing it from core *Fallopia* species. Shade-tolerant climbers (e.g., *F. convolvulus*) have minimal secondary growth (1–3 cell layers) and abundant cortical chlorenchyma (30 - 40% of stem cross-section), supporting photosynthesis in light-limited understories (Desjardins et al., 2023). Disturbance-adapted climbers (e.g., *Pleuropterus multiflorus*) develop irregular secondary xylem with localized vessel clusters, optimizing water transport in heterogeneous soil moisture conditions (Zhang et al., 2022a; Cabon et al., 2020). Collectively, these anatomical innovations underscore the evolutionary flexibility of Polygoneae in navigating the diverse ecological niches of the Northern Hemisphere. In contrast to the high-overlap cases, several genus pairs demonstrated remarkably low niche overlap (e.g., 0.071 between *Atraphaxis* and *Pleuropterus*; 0.041 between *Muehlenbeckia* and *Pleuropterus*). This pronounced niche differentiation likely reflects divergent evolutionary trajectories and adaptation to distinct selective pressures. The consistently low overlap of *Atraphaxis* with most other genera further reinforces its status as a phylogenetically and ecologically distinct lineage within the tribe. These patterns are consistent with the theory of niche differentiation, which holds that reducing ecological overlap minimizes competition and can promote species coexistence and adaptive radiation. Thus, niche divergence likely acted in concert with geographical and reproductive isolation to drive speciation and shape the diversity within Polygoneae.

Our findings collectively support that the radiation of Polygoneae was propelled by a sequence of ecological opportunities generated by Neogene climatic and tectonic shifts. Initially, habitat fragmentation due to aridification and mountain uplift triggered allopatric speciation by isolating populations (e.g., *P. popovii*). Subsequently, the colonization of these newly formed arid habitats was enabled by rapid adaptive evolution, including the emergence of xeromorphic wood syndromes and molecular adaptations in stress-related genes such as *psaI* (Ka/Ks = 1.40). Furthermore, contrasting evolutionary dynamics in key traits dictated differential dispersal success across biogeographic corridors: conserved life-history traits served as dispersal filters, while labile floral traits promoted local adaptation. These combined effects thereby shaped the present-day distribution and diversity of the tribe (perianth number K = 0.23; inflorescence structure K = 0.29). Consistent with the classic ecological opportunity model, abiotic changes created heterogeneous landscapes that drove both geographic isolation and adaptive diversification, a pattern reflected in this progression. The radiation pattern of Polygoneae shares striking parallels with other temperate plant groups diversifying in response to Neogene climatic transitions, yet exhibits unique features that distinguish it as an exceptional model system. The independent evolution of woodiness in multiple Polygoneae lineages parallels the convergent evolution of sclerophylly in Mediterranean *Quercus* (Hipp et al., 2020) and *Ceanothus* (Ackerly, 2009), suggesting predictable adaptive responses to increasing aridity across temperate regions. Similarly, the SW China-Central Asia corridor facilitated dispersal in Polygoneae woody lineages, mirroring its role in Nitrariaceae (Sha et al., 2014) and highlighting its function as an arid bridge for drought-adapted taxa during the Miocene.

### Environmental filtering and life-form selection across three primary migration corridors

Analysis of pairwise niche overlap (Schoener’s D) revealed a mosaic of ecological similarity across the ten genera of Polygoneae (**Supplementary Table 11**). High overlap (D > 0.79) was concentrated among *Fallopia*, *Muehlenbeckia*, and *Pleuropterus*, with the *Muehlenbeckia-Pleuropterus* pair exhibiting near−complete niche similarity (D = 0.94). This pattern suggests these genera occupy virtually identical environmental space, which may reflect shared ancestral niches (phylogenetic niche conservatism) or convergent adaptation to similar habitats (**Supplementary Table 12**). In contrast, *Atraphaxis* showed consistently low overlap with multiple genera (e.g., *Polygonella* and *Reynoutria*; D < 0.16), indicating pronounced niche differentiation. When interpreted in a phylogenetic context, high overlap between closely related lineages could signal recent divergence with limited ecological divergence; in contrast, low overlap between distant clades may point to long−term isolation or adaptive divergence along distinct environmental axes. Building on the anatomical and ecological divergence described above, we identify three primary migration corridors that have functioned as selective environmental filters, shaping contemporary biogeographic patterns through life-form-specific establishment success (**Figure 5**). Within this anatomical framework, the contrasting strategies of woody versus herbaceous lineages are conceptualized as distinct anatomical syndromes: a xylem-dominated syndrome in woody shrubs, characterized by extensive secondary growth, thick-walled lignified cells, and reduced pith (**Figures 4K, L**); and a pith-dominated syndrome in herbaceous taxa, defined by a dominant parenchymatous pith, limited secondary xylem, and reliance on primary growth and turgor support (**Figures 4S, T**). Each corridor imposed distinct selective pressures that filtered lineages based on their anatomical syndromes, creating the disjunct distributions observed today.

The Southwest China–Central Asia corridor functioned as a strong environmental filter, favoring arid-adapted lineages during the westward expansion of woody shrubs (Xu and Zhang, 2015; Zhang et al., 2022b; Qiu et al., 2011). Paleoclimatic reconstructions indicate progressive aridification from the middle Miocene (15 Ma) through the Pliocene (3 Ma), driven by Tianshan uplift and the retreat of Paratethys Sea (Zhuang et al., 2011), which reduced mean annual precipitation from >400 mm to <150 mm across the region. Anatomical analyses reveal that woody taxa successfully established in this corridor consistently exhibit extreme xeromorphic features: pith reduction to <15% of stem diameter, secondary xylem exceeding 12 cell layers, and complete periderm development (**Figures 4K, L**). The absence of herbaceous taxa in contemporary Central Asian populations contrasts sharply with their documented presence in early Miocene fossil assemblages from the Junggar Basin (23–16 Ma) (Manchester and O’Leary, 2010; Zhou et al., 1999), which was robustly supported by high model accuracy (AUC > 0.85), indicating progressive exclusion during Plio-Pleistocene aridification. Characterized by large pith cavities (55 - 70%) and minimal secondary growth, the pith-dominated anatomical syndrome is adaptive in temperate mesic environments (MAP >400 mm), but maladaptive under hyperarid conditions (MAP <150 mm). Large pith cavities, with their high proportion of parenchymatous tissue, are prone to rapid desiccation and cellular collapse under extreme negative water potential (Mauseth, 2006). The vulnerability of this structural syndrome to freeze-thaw cycles during the cold Central Asian winters (Sun et al., 2015; Stewart et al., 2009) thus functioned as a decisive environmental filter. By the late Pliocene (~3 Ma), this filtering process effectively barred herbaceous lineages from further expansion into these arid habitats, confining their subsequent distributions primarily to eastern mesic regions (**Figure 5**).

The Southwest–Northeast corridor, in stark contrast to the xeric filter of the Southwest China–Central Asia corridor, functioned as a mesic refugium that maintained stable conditions for herbaceous lineages throughout Quaternary glacial-interglacial cycles (2.6 Ma–present). The topographic complexity of this corridor (elevation range 500-3,000 m), combined with high orographic precipitation (annual precipitation 800-1,200 mm), created a mosaic of microhabitats across elevational gradients, providing thermal and hydrological buffering against regional climatic extremes (Cao et al., 2023; Schuster et al., 2013). This heterogeneity enabled herbaceous populations to persist through multiple glacial cycles via short-distance elevational migrations (typically <500 m) rather than long-distance latitudinal shifts (Matthew Ogburn and Edwards, 2015). This pattern suggests competitive exclusion by rapidly expanding herbaceous taxa in mesic environments, where their shorter life cycles (1–2 years) and greater phenological flexibility allowed them to exploit brief interglacial windows more efficiently than woody species with longer generation times (>10 years) and high lignification costs (Pratt et al., 2007; Xie et al., 2018). The third corridor, extending along the Quaternary refugial belt of the Hengduan-Qinling-Yanshan mountains, primarily facilitated liana dispersal through topographically complex terrain (Cao et al., 2025; Mao et al., 2021). This corridor’s filtering was driven by humidity gradients (annual precipitation >1,500 mm) and support availability (host tree density), favoring climbers with semi-woody stems and adventitious roots. The intermediate anatomical features observed in climbing species (**Figures 4M–R**), characterized by moderate pith development (30 - 50% of stem diameter) and plastic secondary growth (1–14 cell layers), reflect adaptations to mesic forest-edge habitats. Topographic complexity provided stable microhabitats during glacial periods, enabling lianas to persist in refugia and subsequently colonize adjacent mountain systems (Pandey, 2021; Desjardins et al., 2023). Our integrated anatomical, ecological, and biogeographic analyses reveal that Polygoneae diversification has been fundamentally shaped by a life-form-specific trade-off between dispersal capacity and environmental tolerance, reflecting divergent evolutionary strategies forged through millions of years of corridor-mediated filtering.

## Data Availability

The datasets presented in this study can be found in online repositories. The names of the repository/repositories and accession number(s) can be found in the article/**Supplementary Material**.

## Supplementary material

Supplementary Figure 1 Phylogenetic relationships within Polygoneae inferred from Bayesian analysis of chloroplast genome data. Posterior probabilities are shown at nodes (only values ≥0.80 displayed for clarity).

Supplementary Figure 2 Maximum likelihood phylogenetic analysis of Polygoneae based on multiple chloroplast DNA fragments combined with complete plastome data. Numbers next to each node denote bootstrap support values.

Supplementary Figure 3 East Asia is inferred as the most probable ancestral area for Polygoneae based on ancestral range reconstruction.

Supplementary Figure 4 Time-calibrated phylogenetic tree of Polygonaceae showing divergence times among major lineages.
