# Supplementary figures and images for: Plastome phylogenomics unveils an East Asian origin and climatic niche-driven radiation of the temperate tribe Polygoneae (Polygonaceae)
Source: Front Plant Sci. 2026 Mar 18;17:1792990. doi: 10.3389/fpls.2026.1792990 (PMC13038949; doi:10.3389/fpls.2026.1792990)

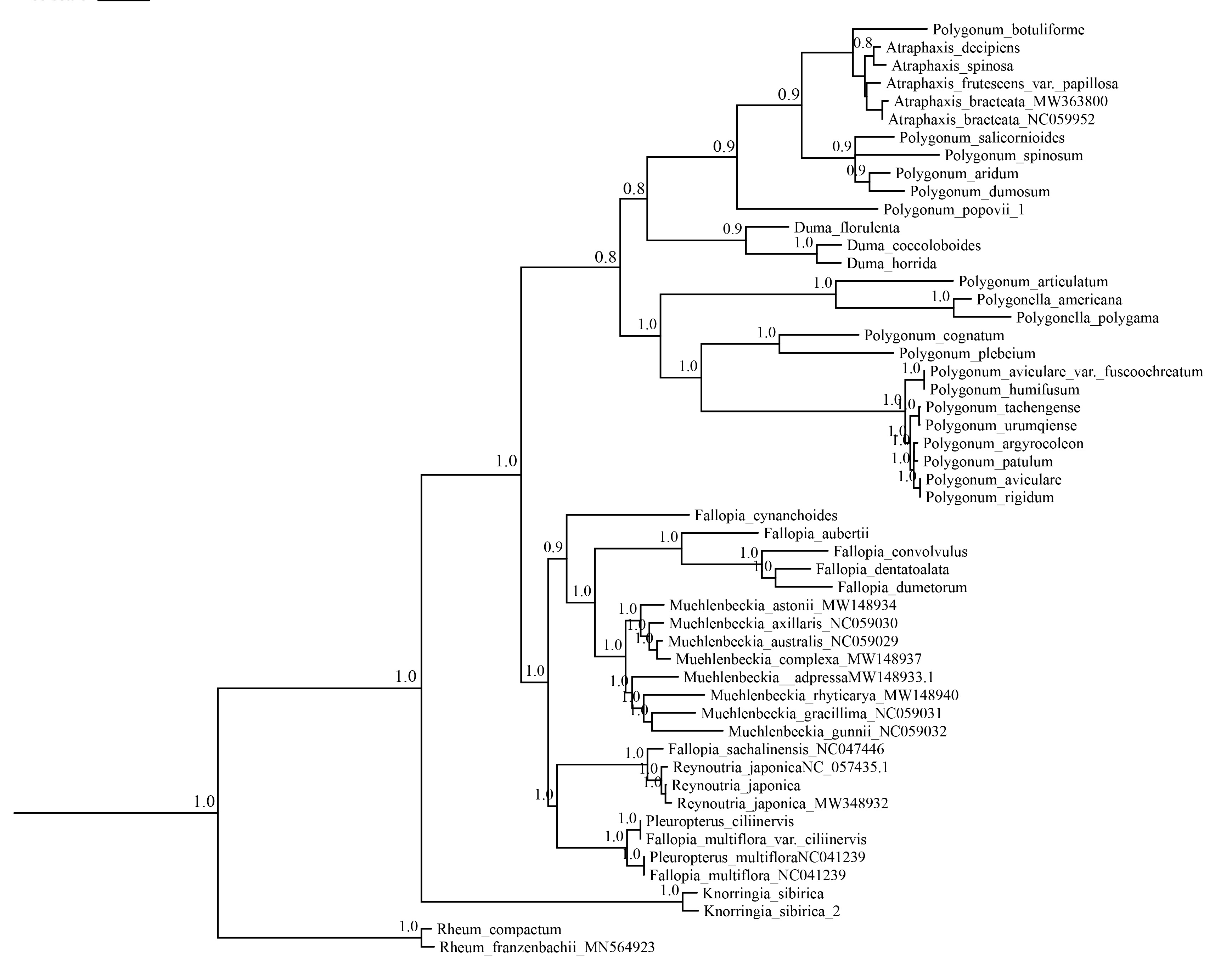

Supplement: Supplementary Figure 1 — Phylogenetic relationships within Polygoneae inferred from Bayesian analysis of chloroplast genome data. Posterior probabilities are shown at nodes (only values ≥0.80 displayed for clarity). [file Image1.jpeg]

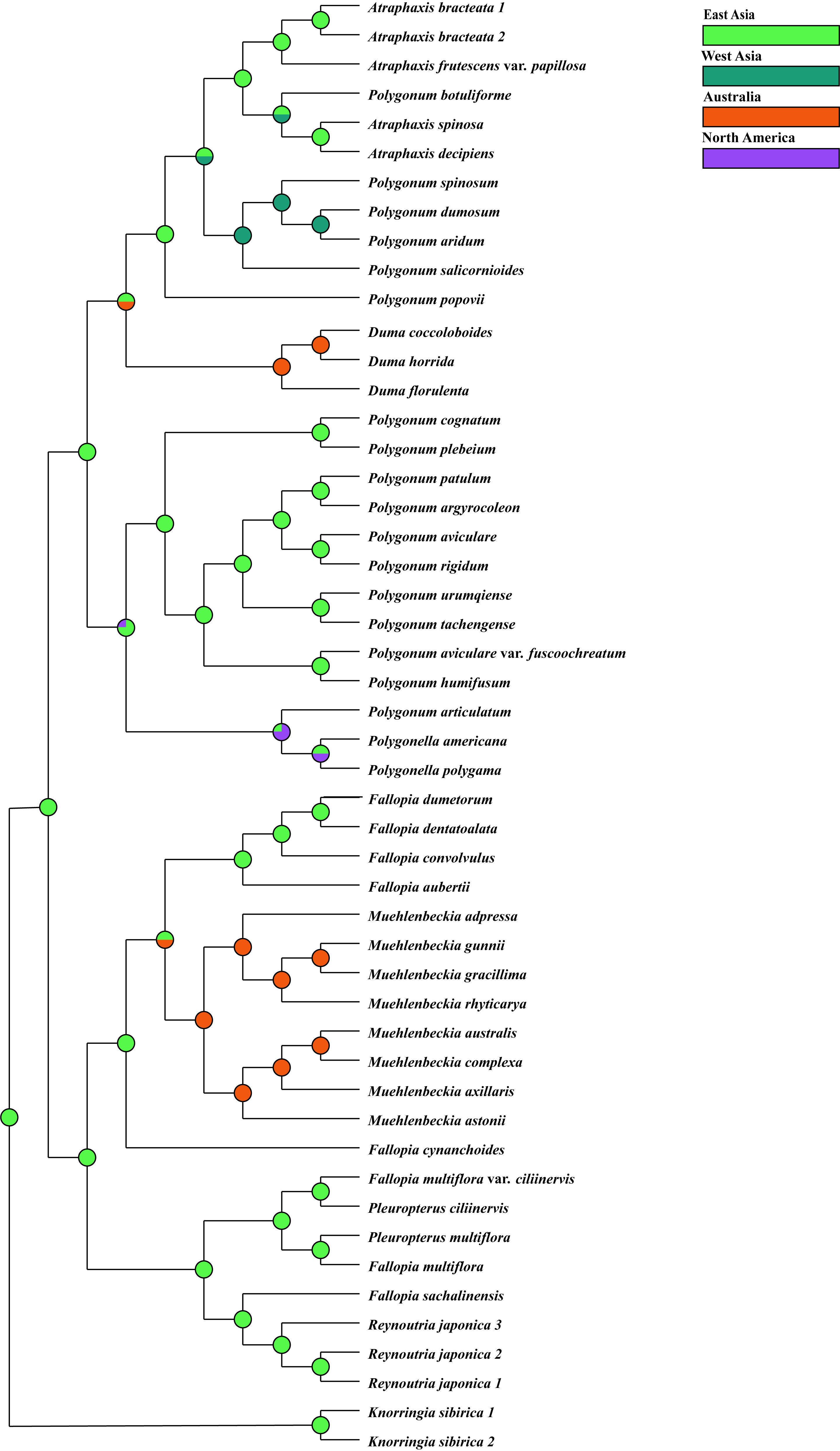

Supplement: Supplementary Figure 3 — East Asia is inferred as the most probable ancestral area for Polygoneae based on ancestral range reconstruction. [file Image3.jpeg]

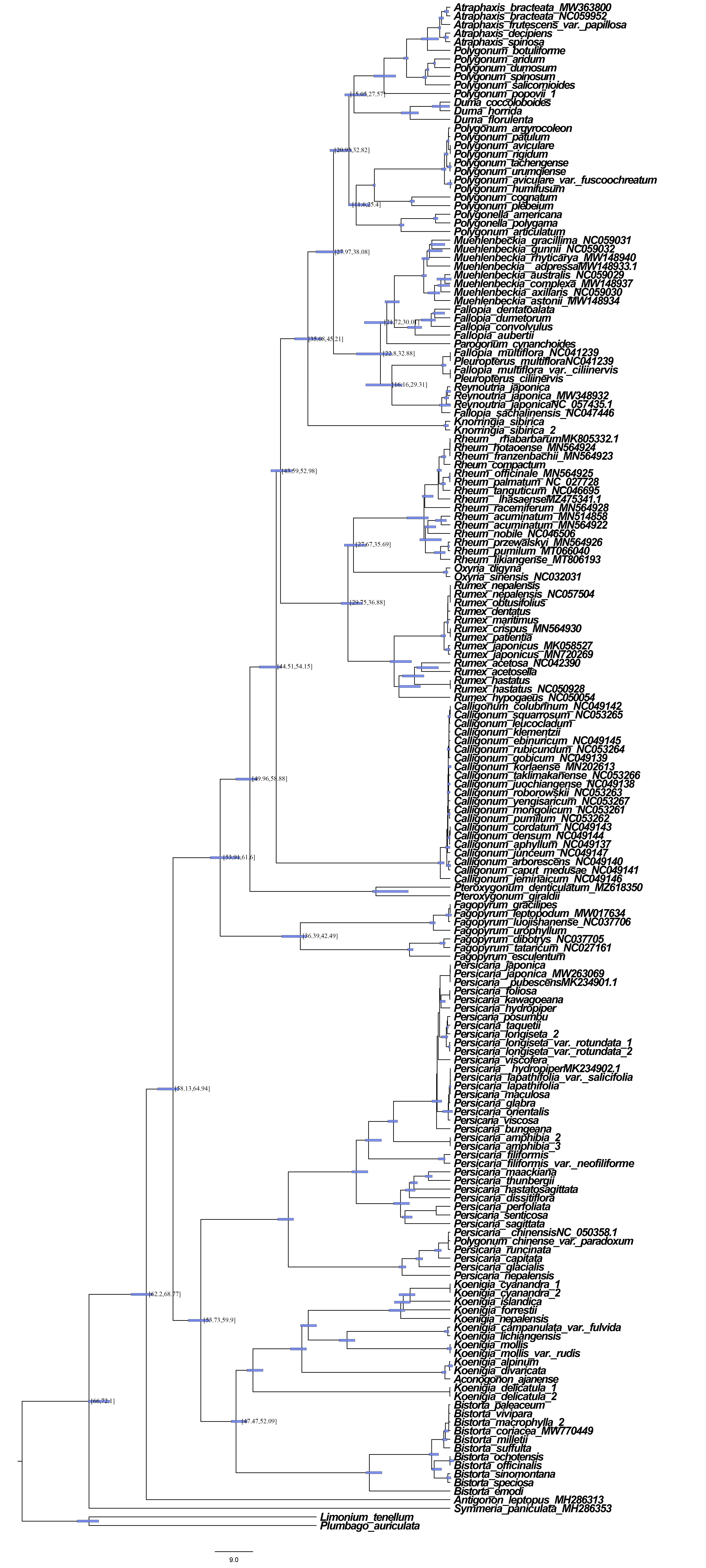

Supplement: Supplementary Figure 4 — Time-calibrated phylogenetic tree of Polygonaceae showing divergence times among major lineages. [file Image4.jpeg]
