# Supplementary material for: Plastome phylogenomics unveils an East Asian origin and climatic niche-driven radiation of the temperate tribe Polygoneae (Polygonaceae)
Source: Front Plant Sci. 2026 Mar 18;17:1792990. doi: 10.3389/fpls.2026.1792990 (PMC13038949; doi:10.3389/fpls.2026.1792990)
Supplement: Supplementary file 5 [file Table1.docx]

**Table S1.** Sampling information for 94 plastomes sequenced from field-collected samples.

| **Species** | **Voucher specimen** | **Locality** | **Coordinate** | **Accession number** |
| --- | --- | --- | --- | --- |
| *Atraphaxis frutescens* var. *papillosa* | SD325 | Urumqi, Xinjiang, China | 87°28'E, 43°48'N | ON229542 |
| *Atraphaxis spinosa* | SD324 | Urumqi, Xinjiang, China | 87°61'E, 43°83'N | ON229543 |
| *Bistorta emodi* | SD280 | Lijiang, Yunnan, China | 100°13'E, 27°01'N | MZ573781 |
| *Bistorta macrophylla* | SD429 | Kunming, Yunnan, China | 102°42'E, 25°02'N | ON229544 |
| *Bistorta milletii* | SD283 | Lijiang, Yunnan, China | 100°43'E, 27°38'N | ON229545 |
| *Bistorta ochotensis* | SD279 | Changbaishan, Jilin, China | 128°40'E, 42°10'N | ON229546 |
| *Bistorta officinalis* | SD369 | Zibo, Shandong, China | 118°30'E, 36°17'N | ON229547 |
| *Bistorta paleaceum* | SD249 | Kunming, Yunnan, China | 102°49'E, 24°53'N | ON229603 |
| *Bistorta sinomontana* | SD286 | Lijiang, Yunnan, China | 100°13'E, 27°01'N | ON229548 |
| *Bistorta amplexicaulis* | SD406 | Shangri-La, Yunnan, China | 99°38'E, 27°54'N | ON229549 |
| *Bistorta suffulta* | SD284 | Huangshan, Anhui, China | 118°10'E, 30°70'N | ON229550 |
| *Bistorta vivipara* | SD278 | Urumqi, Xinjiang, China | 116°28'E, 40°56'N | ON229551 |
| *Calligonum klementzii* | SD266 | Qitai, Xinjiang, China | 89°36'E, 44°10'N | ON229552 |
| *Calligonum leucocladum* | SD265 | Qitai, Xinjiang, China | 89°36''E, 44°10'N | ON229553 |
| *Fagopyrum esculentum* | SD271 | Jinan, Shandong, China | 119°57'E, 36°70'N | ON229554 |
| *Fagopyrum gracilipes* | SD270 | Kunming, Yunnan, China | 102°45'E, 25°80'N | ON229555 |
| *Fagopyrum urophyllum* | SD268 | Xishuangbanna, Yunnan, China | 101°15'E, 21°55'N | ON229556 |
| *Fallopia aubertii* | SD327 | Jinan, Shandong, China | 117°2'E, 36°38'N | ON229557 |
| *Fallopia convolvulus* | SD326 | Tumen, Jilin, China | 129°51'E, 42°58'N | ON229558 |
| *Fallopia dentatoalata* | SD328 | Tumen, Jilin, China | 129°51'E, 42°58'N | ON229559 |
| *Fallopia dumetorum* | SD329 | Nanyang, Henan, China | 119°56'E, 36°57'N | ON229560 |
| *Fallopia multiflora* var. *ciliinervis* | SD331 | Kunming, Yunnan, China | 102°45'E, 25°80'N | ON229561 |
| *Knorringia sibirica* 1 | SD332 | Qingdao, Shandong, China | 120°14'E, 35°58'N | ON229562 |
| *Knorringia sibirica* 2 | SD651 | Kunming, Yunnan, China | 99°44'E, 27°50'N | ON229563 |
| *Koenigia ajanense* | SD315 | Deqin, Yunnan, China | 99°70'E, 28°17'N | MZ573782 |
| *Koenigia alpinum* | SD277 | Taian, Shandong, China | 117°90'E, 36°13'N | MZ573783 |
| *Koenigia campanulata* var. *fulvida* | SD401 | Dali, Yunnan, China | 100°60'E, 24°41'N | MZ573784 |
| *Koenigia cyanandra* 1 | SD409 | Deqin, Yunnan, China | 99°40'E, 28°20'N | MZ573785 |
| *Koenigia cyanandra* 2 | SD428 | Kunming, Yunnan, China | 102°44'E, 25°80'N | MZ573786 |
| *Koenigia delicatula* 1 | SD275 | Lijiang, Yunnan, China | 100°43'E, 27°38'N | MZ573787 |
| *Koenigia delicatula* 2 | SD410 | Deqin, Yunnan, China | 99°40'E, 28°20'N | MZ573788 |
| *Koenigia divaricata* | SD389 | Haerbin, Heilongjiang, China | 126°58'E, 45°90′N | MZ573789 |
| *Koenigia forrestii* | SD408 | Deqin, Yunnan, China | 99°40'E, 28°20'N | MZ573790 |
| *Koenigia islandica* | SD272 | Deqin, Yunnan, China | 99°70'E, 28°17'N | MZ573791 |
| *Koenigia lichiangensis* | SD402 | Wei, Yunnan, China | 99°19'E, 27°20'N | MZ573792 |
| *Koenigia mollis* | SD400 | Nanjian, Yunnan, China | 100°26'E, 24°51'N | MZ573793 |
| *Koenigia mollis* var. *rudis* | SD304 | Napo, Guangxi, China | 120°40'E, 24°80'N | MZ573794 |
| *Koenigia nepalensis* | SD273 | Zhongdian, Yunnan, China | 99°42'E, 27°50'N | MZ573795 |
| *Oxyria digyna* | SD377 | Deqin, Yunnan, China | 99°70'E, 28°17'N | ON229564 |
| *Parogonum cynanchoides* | SD861 | Bijie, Guizhou, China | 106°04'E, 27°01'N | PX704451 |
| *Persicaria amphibia* 1 | SD638 | Qingdao, Shandong, China | 120°28'E, 36°06'N | ON229565 |
| *Persicaria amphibia* 2 | SD637 | Zhangqiu, Shandong, China | 117°23'E, 36°51'N | ON229566 |
| *Persicaria bungeana* | SD376 | Pingdingshan, Henan, China | 112°54'E, 33°44'N | ON229567 |
| *Persicaria capitata* | SD404 | Dali, Yunnan, China | 100°27'E, 24°49'N | ON229568 |
| *Persicaria chinense* var. *paradoxum* | SD411 | Dali, Yunnan, China | 100°70'E, 25°43'N | ON229600 |
| *Persicaria dissitiflora* | SD313 | Taian, Shandong, China | 117°80'E, 36°11'N | ON229569 |
| *Persicaria filiformis* | SD250 | Kunming, Yunnan, China | 102°49'E, 24°53'N | ON229570 |
| *Persicaria neofiliformis* | SD314 | Jiujiang, Jiangxi, China | 115°59'E, 29°33'N | ON229571 |
| *Persicaria foliosa* | SD298 | Antu, Jilin, China | 129°20'E, 43°10'N | ON229572 |
| *Persicaria glabra* | SD291 | Baise, Guangxi, China | 107°16'E, 23°57'N | ON229573 |
| *Persicaria glacialis* | SD306 | Lijiang, Yunnan, China | 116°34'E, 40°26'N | ON229574 |
| *Persicaria hastatosagittata* | SD403 | Diqing, Yunnan, China | 99°19'E, 27°20'N | ON229575 |
| *Persicaria hydropiper* | SD293 | Rizhao, Shandong, China | 119°24'E, 35°41'N | ON229576 |
| *Persicaria japonica* | SD288 | Qingdao, Shandong, China | 120°35'E, 36°80'N | ON229577 |
| *Persicaria kawagoeana* | SD299 | Guangzhou, Guangdong, China | 113°22'E, 23°70'N | ON229578 |
| *Persicaria lapathifolia* | SD292 | Shangzhi, Heilongjiang, China | 128°25'E, 45°10'N | ON229579 |
| *Persicaria lapathifolia* var*. salicifolia* | SD646 | Qingdao, Shandong, China | 120°28'E, 36°06'N | ON229580 |
| *Persicaria longiseta* | SD662 | Chengdu, Sichuan, China | 104°02'E, 30°38'N | ON229581 |
| *Persicaria longiseta* var. *rotundata* 1 | SD658 | Chengdu, Sichuan, China | 104°02'E, 30°38'N | ON229582 |
| *Persicaria longiseta* var. *rotundata* 2 | SD659 | Shangzhi, Heilongjiang, China | 128°23'E, 44°57'N | ON229583 |
| *Persicaria maackiana* | SD307 | Shangzhi, Heilongjiang, China | 116°13'E, 39°47'N | ON229584 |
| *Persicaria maculosa* | SD289 | Jinan, Shandong, China | 117°31'E, 36°41'N | ON229585 |
| *Persicaria nepalensis* | SD303 | Taian, Shandong, China | 117°90'E, 36°13'N | ON229586 |
| *Persicaria orientalis* | SD294 | Jinan, Shandong, China | 119°57'E, 36°70'N | ON229587 |
| *Persicaria perfoliata* | SD370 | Zibo, Shandong, China | 118°30'E, 36°17'N | ON229588 |
| *Persicaria posumbu* | SD295 | Baise, Guangxi, China | 105°48'E, 23°19'N | ON229589 |
| *Persicaria runcinata* | SD302 | Leshan, Sichuan, China | 103°29'E, 29°36'N | ON229590 |
| *Persicaria sagittata* | SD310 | Taian, Shandong, China | 116°60'E, 39°56'N | ON229591 |
| *Persicaria senticosa* | SD309 | Tumen, Jilin, China | 129°51'E, 42°58'N | ON229592 |
| *Persicaria taquetii* | SD297 | Jiujiang, Jiangxi, China | 116°12'E, 29°32'N | ON229593 |
| *Persicaria thunbergii* | SD308 | Chengdu, Sichuan, China | 103°37'E, 31°01'N | ON229594 |
| *Persicaria viscofera* | SD287 | Jinan, Shandong, China | 119°57'E, 36°70'N | ON229595 |
| *Persicaria viscosa* | SD300 | Shangzhi, Heilongjiang, China | 128°25'E, 45°10'N | ON229596 |
| *Polygonum argyrocoleon* | SD316 | Tacheng, Xinjiang, China | 103°33'E, 29°25'N | ON229597 |
| *Polygonum aviculare* | SD317 | Lijiang, Yunnan, China | 100°12'E, 27°01'N | ON229598 |
| *Polygonum aviculare* var. *fusco-ochreatum* | SD636 | Huichun, Jilin, China | 130°21'E, 42°51'N | ON229599 |
| *Polygonum cognatum* | SD319 | Kuche, Xinjiang, China | 116°24'E, 39°54'N | ON229601 |
| *Polygonum humifusum* | SD643 | Huichun, Jilin, China | 130°21'E, 42°51'N | ON229602 |
| *Polygonum patulum* | SD321 | Urumqi, Xinjiang, China | 87°18'E, 43°29'N | ON229604 |
| *Polygonum plebeium* | SD322 | Jinan, Shandong, China | 116°57'E, 36°30'N | ON229605 |
| *Polygonum popovii* | SD648 | Tianshan, Xinjiang, China | 83°5E′, 42°8′N | PV670016 |
| *Polygonum rigidum* | SD323 | Taian, Shandong, China | 121°25'E, 25°30'N | ON229606 |
| *Polygonum tachengense* | SD640 | Tacheng, Xinjiang, China | 82°58'E, 46°44'N | ON229607 |
| *Polygonum urumqiense* | SD641 | Urumqi, Xinjiang, China | 88°18'E, 43°21'N | ON229608 |
| *Pteroxygonum giraldii* | SD635 | Jincheng, Shanxi, China | 109°08'E, 34°11'N | ON229609 |
| *Reynoutria japonica* | SD330 | Taian, Shandong, China | 121°25'E, 25°30'N | ON229610 |
| *Rheum compactum* | SD258 | Qingdao, Shandong, China | 87°10'E, 43°15'N | ON229611 |
| *Rumex acetosella* | SD259 | Qingdao, Shandong, China | 120°40'E, 36°14'N | ON229612 |
| *Rumex dentatus* | SD261 | Jinan, Shandong, China | 116°38'E, 40°19'N | ON229613 |
| *Rumex hastatus* | SD260 | Deqin, Yunnan, China | 98°55'E, 28°28'N | ON229614 |
| *Rumex maritimus* | SD262 | Mudanjiang, Heilongjiang, China | 128°25'E, 45°10'N | ON229615 |
| *Rumex nepalensis* | SD251 | Qingdao, Shandong, China | 120°38'E, 36°06'N | MT457825 |
| *Rumex obtusifolius* | SD120 | Qingdao, Shandong, China | 120°63'E, 36°15'N | ON229616 |
| *Rumex patientia* | SD119 | Qingdao, Shandong, China | 120°28'E, 36°13'N | ON229617 |
