# Supplementary material for: Plastome phylogenomics unveils an East Asian origin and climatic niche-driven radiation of the temperate tribe Polygoneae (Polygonaceae)
Source: Front Plant Sci. 2026 Mar 18;17:1792990. doi: 10.3389/fpls.2026.1792990 (PMC13038949; doi:10.3389/fpls.2026.1792990)
Supplement: Supplementary file 6 [file Table2.docx]

**Table S2.** Information on downloading sequences from NCBI.

| **Species** | **Accession number** |
| --- | --- |
| *Antigonon leptopus* | MH286313 |
| *Atraphaxis_decipiens* | NC070100 |
| *Atraphaxis bracteata*1 | MW363800 |
| *Atraphaxis bracteata*2 | NC059952 |
| *Bistorta coriacea* | MW770449 |
| *Calligonum aphyllum* | NC049137 |
| *Calligonum arborescens* | NC049140 |
| *Calligonum caput-medusae* | NC049141 |
| *Calligonum colubrinum* | NC049142 |
| *Calligonum cordatum* | NC049143 |
| *Calligonum densum* | NC049144 |
| *Calligonum ebinuricum* | NC049145 |
| *Calligonum gobicum* | NC049139 |
| *Calligonum jeminaicum* | NC049146 |
| *Calligonum junceum* | NC049147 |
| *Calligonum juochiangense* | NC049138 |
| *Calligonum korlaense* | MN202613 |
| *Calligonum mongolicum* | NC053261 |
| *Calligonum pumilum* | NC053262 |
| *Calligonum roborowskii* | NC053263 |
| *Calligonum rubicundum* | NC053264 |
| *Calligonum squarrosum* | NC053265 |
| *Calligonum taklimakanense* | NC053266 |
| *Calligonum yengisaricum* | NC053267 |
| *Fagopyrum dibotrys* | NC037705 |
| *Fagopyrum leptopodum* | MW017634 |
| *Fagopyrum luojishanense* | NC037706 |
| *Fagopyrum tataricum* | NC027161 |
| *Limonium_tenellum* | NC041279. |
| *Muehlenbeckian adpressa* | MW148933 |
| *Muehlenbeckia astonii* | MW148934 |
| *Muehlenbeckia australis* | NC059029 |
| *Muehlenbeckia axillaris* | NC059030 |
| *Muehlenbeckia complexa* | MW148937 |
| *Muehlenbeckia gracillima* | NC059031 |
| *Muehlenbeckia gunnii* | NC059032 |
| *Muehlenbeckia rhyticarya* | MW148940 |
| *Oxyria sinensis* | NC032031 |
| *Persicaria japonica* 2 | MW263069 |
| *Persicaria chinensis* | *NC050358* |
| *Persicaria hydropiper* | *MK234902* |
| *Persicaria pubescens* | *MK234901* |
| *Pleuropterus multiflora* | *NC041239* |
| *Plumbago_auriculata* | MH286308 |
| *Pteroxygonum denticulatum* | MZ618350 |
| *Reynoutria_japonica* | NC057435 |
| *Reynoutria japonica* 2 | MW348932 |
| *Reynoutria multiflora* | NC041239 |
| *Reynoutria sachalinensis* | NC047446 |
| *Reynoutria multiflora* | NC041239 |
| *Rheum lhasaense* | MZ475341 |
| *Rheum rhabarbarum* | MK805332 |
| *Rheum acuminatum* 1 | MN514858 |
| *Rheum acuminatum* 2 | MN564922 |
| *Rheum franzenbachii* | MN564923 |
| *Rheum hotaoense* | MN564924 |
| *Rheum likiangense* | MT806193 |
| *Rheum nobile* | NC046506 |
| *Rheum officinale* | MN564925 |
| *Rheum palmatum* | NC027728 |
| *Rheum przewalskyi* | MN564926 |
| *Rheum pumilum* | MT066040 |
| *Rheum racemiferum* | MN564928 |
| *Rheum tanguticum* | NC046695 |
| *Rumex acetosa* | NC042390 |
| *Rumex crispus* | MN564930 |
| *Rumex hastatus* 2 | NC050928 |
| *Rumex hypogaeus* | NC050054 |
| *Rumex japonicus* 1 | MK058527 |
| *Rumex japonicus* 2 | MN720269 |
| *Rumex nepalensis* 1 | NC057504 |
| *Symmeria paniculata* | MH286353 |
| *Duma coccoloboides (ndhF)* | JF831243 |
| *Duma coccoloboides* (*trnK*) | JF831264 |
| *Duma florulenta* (*ndhF*) | JF831244 |
| *Duma florulenta* (*trnK*) | JF831265 |
| *Duma florulenta* (*trnV-ndhC*) | JF831323 |
| *Duma horrida* (*ndhF*) | JF831245 |
| *Duma horrida* (*trnK*) | JF831232 |
| *Duma horrida* (*trnV-ndhC*) | JF831324 |
| *Polygonella americana* (*matK*) | GQ206202 |
| *Polygonella americana* (*ndhF*) | GQ206289 |
| *Polygonella americana* (*rbcL*) | GQ206226 |
| *Polygonella articulata* (*ndhF*) | GQ206290 |
| *Polygonella articulata* (*rbcL*) | EF653760 |
| *Polygonella polygamum* (*rbcL*) | KJ773779 |
| *Polygonella polygamum* (*matK*) | KJ773035 |
| *Polygonum aridum* (*matK*) | AB976667 |
| *Polygonum articulatum* (*rpl32-trnL*) | AB976693 |
| *Polygonum articulatum* (*ndhF*) | GQ206290 |
| *Polygonum articulatum* (*rbcL*) | MK526393 |
| *Polygonum botuliforme* (*matK*) | AB976670 |
| *Polygonum botuliforme* (*rpl32-trnL*) | AB976696 |
| *Polygonum dumosum* (*matK*) | AB976671 |
| *Polygonum dumosum* (*rpl32-trnL*) | AB976697 |
| *Polygonum salicornioides* (*matK*) | AB976702 |
| *Polygonum salicornioides* (*rpl32-trnL*) | AB976676 |
| *Polygonum spinosum* (*matK*) | AB976673 |
| *Polygonum spinosum* (*rpl32-trnL*) | AB976699 |
