# Supplementary material for: Plastome phylogenomics unveils an East Asian origin and climatic niche-driven radiation of the temperate tribe Polygoneae (Polygonaceae)
Source: Front Plant Sci. 2026 Mar 18;17:1792990. doi: 10.3389/fpls.2026.1792990 (PMC13038949; doi:10.3389/fpls.2026.1792990)
Supplement: Supplementary file 7 [file Table3.docx]

**Table S3.** Details of the morphological traits used for analysis.

| **Species/Trait** | **Leaf venation** | **Tepal venation** | **Leaf abaxial epidermal cell shape** | **Stomata type** | **Fruit surface sculpture** | **Exocarp structure** | **Stamens**  **Number** |
| --- | --- | --- | --- | --- | --- | --- | --- |
| ***Fallopia aubertii*** | palmate venation | pinnate venation | irregular | mixture of both | neoplastic | dichotomous to trichotomous | 8 |
| ***Fallopia convolvulus*** | palmate venation | pinnate venation | irregular | mixture of both | neoplastic | dichotomous to trichotomous | 8 |
| ***Fallopia dentatoalata*** | palmate venation | pinnate venation | irregular | mixture of both | neoplastic | dichotomous to trichotomous | 8 |
| ***Fallopia dumetorum*** | palmate venation | pinnate venation | irregular | mixture of both | mound | dichotomous to trichotomous | 8 |
| ***Reynoutria japonica*** | pinnate venation | pinnate venation | irregular | anisocytic | pits | dichotomous | 8 |
| ***Reynoutria multiflora*** | palmate venation | pinnate venation | polygonal | anisocytic | undulating | dichotomous to trichotomous | 8 |
| ***Reynoutria multiflora var. ciliinervis*** | palmate venation | pinnate venation | polygonal | anisocytic | undulating | dichotomous to trichotomous | 8 |
| ***Polygonum rigidum*** | pinnate venation | pinnate venation | polygonal | anisocytic | neoplastic | dichotomous,trichotomous,  dendritic, or absent | 8 |
| ***Polygonum aviculare*** | pinnate venation | pinnate venation | polygonal | anisocytic | neoplastic | dichotomous,trichotomous,  dendritic, or absent | 8 |
| ***Polygonum argyrocoleon*** | pinnate venation | pinnate venation | polygonal | anisocytic | smooth | dichotomous,trichotomous,  dendritic, or absent | 8 |
| ***Polygonum patulum*** | pinnate venation | pinnate venation | polygonal | anisocytic | neoplastic | dichotomous,trichotomous,  dendritic, or absent | 8 |
| ***Polygonum tachengense*** | pinnate venation | pinnate venation | polygonal | anisocytic | warty | dichotomous,trichotomous,  dendritic, or absent | 8 |
| ***Polygonum urumqiense*** | pinnate venation | pinnate venation | irregular | anisocytic | neoplastic | dichotomous,trichotomous,  dendritic, or absent | 8 |
| ***Polygonum humifusum*** | pinnate venation | pinnate venation | polygonal | anisocytic | neoplastic | dichotomous,trichotomous,  dendritic, or absent | 8 |
| ***Polygonum aviculare var. fusco-ochreatum*** | pinnate venation | pinnate venation | polygonal | anisocytic | neoplastic | dichotomous,trichotomous,  dendritic, or absent | 8 |
| ***Polygonum plebeium*** | pinnate venation | pinnate venation | polygonal | anisocytic | smooth | dichotomous,trichotomous,  dendritic, or absent | 5 |
| ***Polygonum cognatum*** | pinnate venation | pinnate venation | polygonal | anisocytic | smooth | dichotomous,trichotomous,  dendritic, or absent | 8 |
| ***Knorringia sibirica*** | pinnate venation | pinnate venation | polygonal | anisocytic | pits | dichotomous | 7, 8 |
| ***Pteroxygonum denticulatum*** | palmate venation | 3-basinerved | irregular | mixture of both | / | absent | 8 |
| ***Pteroxygonum giraldii*** | palmate venation | 3-basinerved | irregular | mixture of both | neoplastic | absent | 8 |
| ***Fagopyrum leptopodum*** | palmate venation | pinnate venation | irregular | mixture of both | neoplastic | absent | 8 |
| ***Fagopyrum gracilipes*** | palmate venation | pinnate venation | irregular | mixture of both | neoplastic | absent | 8 |
| ***Fagopyrum urophyllum*** | palmate venation | pinnate venation | irregular | mixture of both | neoplastic | absent | 8 |
| ***Fagopyrum dibotrys*** | palmate venation | 3-basinerved | irregular | mixture of both | reticulation | absent | 8 |
| ***Fagopyrum tataricum*** | palmate venation | 3-basinerved | irregular | mixture of both | reticulation | absent | 8 |
| ***Fagopyrum esculentum*** | palmate venation | 3-basinerved | irregular | mixture of both | reticulation | absent | 8 |
| ***Persicaria taquetii*** | pinnate venation | 3-basinerved | irregular | paracytic | reticulation | dichotomous | 7 |
| ***Persicaria posumbu*** | pinnate venation | 3-basinerved | irregular | paracytic | brain stripe | dichotomous | 8 |
| ***Persicaria longiseta*** | pinnate venation | 3-basinerved | irregular | paracytic | brain stripe | dichotomous | 6, 8 |
| ***Persicaria longiseta var. rotundata*** | pinnate venation | 3-basinerved | irregular | paracytic | reticulation | dichotomous | 6, 8 |
| ***Persicaria foliosa*** | pinnate venation | 3-basinerved | irregular | paracytic | pits | dichotomous | 5 |
| ***Persicaria kawagoeana*** | pinnate venation | 3-basinerved | irregular | paracytic | reticulation | dichotomous | 5, 6 |
| ***Persicaria hydropiper*** | pinnate venation | 3-basinerved | irregular | paracytic | reticulation | dichotomous | 6, 8 |
| ***Persicaria japonica*** | pinnate venation | 3-basinerved | irregular | paracytic | brain stripe | dichotomous | 8 |
| ***Persicaria viscofera*** | pinnate venation | 3-basinerved | irregular | anisocytic | pits | dichotomous | 7, 8 |
| ***Persicaria lapathifolia* var*. salicifolia*** | pinnate venation | 3-basinerved | irregular | anisocytic | pits | dichotomous | 6 |
| ***Persicaria lapathifolia*** | pinnate venation | 3-basinerved | irregular | anisocytic | pits | dichotomous | 6 |
| ***Persicaria maculosa*** | pinnate venation | 3-basinerved | irregular | anisocytic | brain stripe | dichotomous | 6, 7 |
| ***Persicaria glabra*** | pinnate venation | 3-basinerved | irregular | paracytic | folds | dichotomous | 6, 8 |
| ***Persicaria orientalis*** | pinnate venation | 3-basinerved | irregular | mixture of both | brain stripe | dichotomous | 7 |
| ***Persicaria viscosa*** | pinnate venation | 3-basinerved | irregular | anisocytic | warty | dichotomous | 8 |
| ***Persicaria bungeana*** | pinnate venation | 3-basinerved | irregular | anisocytic | warty | dichotomous | 7, 8 |
| ***Persicaria amphibia*** | pinnate venation | 3-basinerved | polygonal | anisocytic | pits | dichotomous | 5 |
| ***Persicaria neofiliformis*** | pinnate venation | 3-basinerved | irregular | anisocytic | pits | absent | 5 |
| ***Persicaria filiformis*** | pinnate venation | 3-basinerved | irregular | anisocytic | pits | absent | 5 |
| ***Persicaria maackiana*** | palmate venation | 3-basinerved | irregular | paracytic | folds | absent | 8 |
| ***Persicaria thunbergii*** | palmate venation | 3-basinerved | irregular | paracytic | folds | absent | 8 |
| ***Persicaria hastatosagittata*** | palmate venation | 3-basinerved | irregular | paracytic | folds | absent | 7, 8 |
| ***Persicaria dissitiflora*** | palmate venation | 3-basinerved | irregular | paracytic | / | absent | 7, 8 |
| ***Persicaria perfoliata*** | palmate venation | 3-basinerved | irregular | paracytic | pits | absent | 8 |
| ***Persicaria senticosa*** | palmate venation | 3-basinerved | irregular | paracytic | mound | absent | 8 |
| ***Persicaria sagittata*** | palmate venation | 3-basinerved | irregular | paracytic | neoplastic | absent | 8 |
| ***Persicaria chinense* var*. paradoxum*** | pinnate venation | 3-basinerved | irregular | mixture of both | reticulation | absent | 8 |
| ***Persicaria runcinata*** | pinnate venation | 3-basinerved | irregular | mixture of both | stellate | absent | 8 |
| ***Persicaria capitata*** | pinnate venation | 3-basinerved | irregular | mixture of both | folds | absent | 8 |
| ***Persicaria glacialis*** | pinnate venation | 3-basinerved | irregular | anomocytic | reticulation | absent | 5 |
| ***Persicaria nepalensis*** | pinnate venation | 3-basinerved | irregular | anomocytic | neoplastic | absent | 5, 6 |
| ***Koenigia cyanandra*** | pinnate venation | 3-basinerved | irregular | mixture of both | warty | dichotomous | 8 |
| ***Koenigia islandica*** | pinnate venation | 3-basinerved | irregular | mixture of both | stellate | absent | 3 |
| ***Koenigia forrestii*** | pinnate venation | 3-basinerved | irregular | mixture of both | stellate | dichotomous | 6, 8 |
| ***Koenigia nepalensis*** | pinnate venation | 3-basinerved | irregular | mixture of both | brain stripe | dichotomous | 3, 4 |
| ***Koenigia campanulata* var*. fulvida*** | pinnate venation | 3-basinerved | irregular | mixture of both | brain stripe | dichotomous | 8 |
| ***Koenigia lichiangensis*** | pinnate venation | 3-basinerved | irregular | mixture of both | stellate | dichotomous | 8 |
| ***Koenigia mollis* var. *rudis*** | pinnate venation | 3-basinerved | irregular | mixture of both | stellate | dichotomous | 8 |
| ***Koenigia mollis*** | pinnate venation | 3-basinerved | irregular | mixture of both | stellate | dichotomous | 8 |
| ***Koenigia divaricata*** | pinnate venation | 3-basinerved | irregular | mixture of both | stellate | dichotomous | 7, 8 |
| ***Koenigia alpinum*** | pinnate venation | 3-basinerved | irregular | mixture of both | clutter | dichotomous | 8 |
| ***Koenigia ajanense*** | pinnate venation | 3-basinerved | irregular | mixture of both | clutter | dichotomous | 8 |
| ***Koenigia delicatula*** | pinnate venation | 3-basinerved | irregular | mixture of both | stellate | dichotomous | 3 |
| ***Bistorta paleaceum*** | pinnate venation | 3-basinerved | irregular | paracytic | stellate | dichotomous | 8 |
| ***Bistorta vivipara*** | pinnate venation | 3-basinerved | irregular | mixture of both | stellate | dichotomous | 8 |
| ***Bistorta macrophylla*** | pinnate venation | 3-basinerved | irregular | mixture of both | stellate | dichotomous | 8 |
| ***Bistorta milletii*** | pinnate venation | 3-basinerved | irregular | / | stellate | dichotomous | 8 |
| ***Bistorta suffulta*** | pinnate venation | 3-basinerved | irregular | mixture of both | stellate | dichotomous | 8 |
| ***Bistorta officinalis*** | pinnate venation | 3-basinerved | irregular | anisocytic | stellate | dichotomous | 8 |
| ***Bistorta ochotensis*** | pinnate venation | 3-basinerved | irregular | mixture of both | stellate | dichotomous | 8 |
| ***Bistorta amplexicaulis*** | pinnate venation | 3-basinerved | irregular | mixture of both | stellate | dichotomous | 8 |
| ***Bistorta sinomontana*** | pinnate venation | 3-basinerved | irregular | anisocytic | stellate | dichotomous | 8 |
| ***Bistorta emodi*** | pinnate venation | 3-basinerved | irregular | paracytic | pits | dichotomous | 8 |

Note: “/” representative cannot be determined.
