# Supplementary material for: Plastome phylogenomics unveils an East Asian origin and climatic niche-driven radiation of the temperate tribe Polygoneae (Polygonaceae)
Source: Front Plant Sci. 2026 Mar 18;17:1792990. doi: 10.3389/fpls.2026.1792990 (PMC13038949; doi:10.3389/fpls.2026.1792990)
Supplement: Supplementary file 8 [file Table4.docx]

**Table S4.** Data used for ancestral trait reconstruction.

| Species | fruit | life | inflorescence | pollen | year | stigma | stamen |
| --- | --- | --- | --- | --- | --- | --- | --- |
| *Atraphaxis_bracteata_MW363800* | triangular | woody | raceme | 3-colporate | perennial | 3 | 8 |
| *Atraphaxis_bracteata_NC059952* | triangular | woody | raceme | 3-colporate | perennial | 3 | 8 |
| *Atraphaxis_decipiens* | triangular | woody | raceme | 3-colporate | perennial | 3 | 8 |
| *Atraphaxis_frutescens_var._papillosa* | triangular | woody | raceme | 3-colporate | perennial | 2, 3 | 6,8 |
| *Atraphaxis_spinosa* | oval | woody | raceme | 3-colporate | perennial | 2, 3 | 6 |
| *Duma_coccoloboides* | triangular | woody | solitary | 3-colporate | perennial | 2, 3 | 6,8 |
| *Duma_florulenta* | triangular | woody | raceme | 3-colporate | perennial | 2, 3 | 6,8 |
| *Duma_horrida* | triangular | woody | solitary | 3-colporate | perennial | 2, 3 | 6,8 |
| *Fallopia_aubertii* | triangular | woody | panicle | 3-colporate | perennial | 3 | 8 |
| *Fallopia_convolvulus* | triangular | herbal | panicle | 3-colporate | annual | 3 | 8 |
| *Fallopia_cynanchoides* | triangular | herbal | panicle | 3-colporate | perennial | 3 | 8 |
| *Fallopia_dentatoalata* | triangular | herbal | raceme | 3-colporate | annual | 3 | 8 |
| *Fallopia_dumetorum* | triangular | herbal | raceme | 3-colporate | annual | 3 | 8 |
| *Fallopia_multiflora_NC041239* | triangular | herbal | panicle | 3-colporate | perennial | 3 | 8 |
| *Fallopia_multiflora_var._ciliinervis* | triangular | herbal | panicle | 3-colporate | perennial | 3 | 8 |
| *Fallopia_sachalinensis_NC047446* | triangular | vine | panicle | 3-colporate | perennial | 3 | 8 |
| *Knorringia_sibirica* | triangular | herbal | panicle | 3-colporate | perennial | 3 | 7,8 |
| *Knorringia_sibirica_2* | triangular | herbal | panicle | 3-colporate | perennial | 3 | 7,8 |
| *Muehlenbeckia__adpressaMW148933.1* | triangular | vine | solitary | pantoporate | perennial | 3 | 8 |
| *Muehlenbeckia_astonii_MW148934* | triangular | vine | solitary | pantoporate | perennial | 3 | 8 |
| *Muehlenbeckia_australis_NC059029* | triangular | vine | solitary | pantoporate | perennial | 3 | 8 |
| *Muehlenbeckia_axillaris_NC059030* | triangular | vine | solitary | pantoporate | perennial | 3 | 8 |
| *Muehlenbeckia_complexa_MW148937* | triangular | vine | solitary | pantoporate | perennial | 3 | 8 |
| *Muehlenbeckia_gracillima_NC059031* | triangular | vine | solitary | pantoporate | perennial | 3 | 8 |
| *Muehlenbeckia_gunnii_NC059032* | triangular | vine | solitary | pantoporate | perennial | 3 | 8 |
| *Muehlenbeckia_rhyticarya_MW148940* | triangular | vine | solitary | pantoporate | perennial | 3 | 8 |
| *Pleuropterus_ciliinervis* | triangular | herbal | panicle | 3-colporate | perennial | 3 | 8 |
| *Pleuropterus_multifloraNC041239* | triangular | herbal | panicle | 3-colporate | perennial | 3 | 8 |
| *Polygonella_americana* | oval | herbal | raceme | 3-colporate | perennial | 3 | 8 |
| *Polygonella_polygama* | oval | herbal | raceme | 3-colporate | perennial | 3 | 8 |
| *Polygonum_argyrocoleon* | triangular | herbal | raceme | 3-colporate | annual | 3 | 7,8 |
| *Polygonum_aridum* | triangular | herbal | solitary | 3-colporate | perennial | 3 | 8 |
| *Polygonum_articulatum* | triangular | herbal | raceme | 3-colporate | perennial | 3 | 8 |
| *Polygonum_aviculare* | triangular | herbal | solitary | 3-colporate | annual | 3 | 8 |
| *Polygonum_aviculare_var._fuscoochreatum* | triangular | herbal | solitary | 3-colporate | annual | 3 | 8 |
| *Polygonum_botuliforme* | oval | herbal | solitary | 3-colporate | perennial | 3 | 8 |
| *Polygonum_cognatum* | triangular | herbal | solitary | 3-colporate | perennial | 3 | 8 |
| *Polygonum_dumosum* | triangular | herbal | raceme | 3-colporate | perennial | 3 | 8 |
| *Polygonum_humifusum* | triangular | herbal | solitary | 3-colporate | annual | 3 | 8 |
| *Polygonum_patulum* | triangular | herbal | raceme | 3-colporate | annual | 3 | 8 |
| *Polygonum_plebeium* | triangular,oval | herbal | solitary | 3-colporate | annual | 2, 3 | 5 |
| *Polygonum_popovii* | triangular | woody | raceme | 3-colporate | perennial | 3 | 8 |
| *Polygonum_rigidum* | triangular | herbal | solitary | 3-colporate | annual | 3 | 8 |
| *Polygonum_salicornioides* | triangular | herbal | solitary | 3-colporate | perennial | 3 | 8 |
| *Polygonum_spinosum* | triangular | herbal | solitary | 3-colporate | perennial | 3 | 8 |
| *Polygonum_tachengense* | triangular | herbal | solitary | 3-colporate | annual | 3 | 8 |
| *Polygonum_urumqiense* | triangular | herbal | solitary | 3-colporate | annual | 3 | 5,6 |
| *Reynoutria_japonica* | triangular | vine | panicle | 3-colporate | perennial | 3 | 8 |
| *Reynoutria_japonica_MW348932* | triangular | vine | panicle | 3-colporate | perennial | 3 | 8 |
| *Reynoutria_japonicaNC_057435.1* | triangular | vine | panicle | 3-colporate | perennial | 3 | 8 |

Note: “/” representative cannot be determined.
