# Supplementary material for: Plastome phylogenomics unveils an East Asian origin and climatic niche-driven radiation of the temperate tribe Polygoneae (Polygonaceae)
Source: Front Plant Sci. 2026 Mar 18;17:1792990. doi: 10.3389/fpls.2026.1792990 (PMC13038949; doi:10.3389/fpls.2026.1792990)
Supplement: Supplementary file 9 [file Table5.docx]

**Table S5.** Comparative analysis of bioclimatic variable contributions in MaxEnt models across multiple genera.

| Genus | bio1 | bio2 | bio3 | bio4 | bio5 | bio6 | bio7 | bio8 | bio9 | bio10 | bio11 | bio12 | bio13 | bio14 | bio15 | bio16 | bio17 | bio18 | bio19 |
| --- | --- | --- | --- | --- | --- | --- | --- | --- | --- | --- | --- | --- | --- | --- | --- | --- | --- | --- | --- |
| *Atraphaxis* | 10.4 | 3.3 | 13 | 9 | 33.3 | 0.1 | 0.3 | 0.2 | 0.8 | 0.1 | 17.2 | 1 | 0.2 | 0.8 | 0.5 | 0.7 | 0 | 8.1 | 1 |
| *Duma* | 0 | 0.7 | 52.4 | 10.6 | 0.1 | 0.6 | 1 | 0.2 | 0.1 | 0.1 | 8.4 | 0.5 | 0 | 19.7 | 2.1 | 0.4 | 2.4 | 0.6 | 0.1 |
| *Fallopia* | 16.3 | 1 | 0 | 0 | 0 | 0 | 0 | 0 | 0 | 0.6 | 19.8 | 21.3 | 0 | 38.9 | 0.1 | 0 | 0.1 | 0.1 | 1.7 |
| *Muehlenbeckia* | 8.2 | 0.1 | 6.2 | 0.7 | 0.1 | 43.9 | 11.4 | 0 | 0.1 | 0 | 1.2 | 0.1 | 0 | 2.8 | 0.2 | 0 | 0 | 0 | 24.8 |
| *Knorringia* | 23.2 | 2.9 | 30.9 | 2.5 | 10.2 | 0.1 | 0.3 | 0.7 | 0.3 | 7.2 | 0.1 | 9.7 | 0.3 | 6.8 | 0.1 | 0 | 0.1 | 1.2 | 3.2 |
| *Polygonum* | 0.3 | 4.3 | 3.9 | 15.6 | 30.7 | 1 | 0.8 | 0.1 | 1.3 | 0.4 | 0.8 | 0.1 | 0.2 | 32.7 | 0.8 | 0.1 | 0.3 | 2 | 4.7 |
| *Polygonella* | 0.9 | 2.1 | 0.8 | 11.1 | 5.4 | 0.2 | 0.6 | 0.7 | 0.5 | 0.7 | 0.3 | 11.9 | 0.2 | 51 | 0.2 | 0.1 | 12.2 | 0.8 | 0.3 |
| *Reynoutria* | 0.6 | 1.6 | 0.1 | 5.4 | 0.8 | 7 | 1.3 | 0 | 0.9 | 0.4 | 7.7 | 5.3 | 0 | 67.4 | 0.1 | 0 | 0 | 0.1 | 1.4 |
| *Parogonum* | 0 | 6.8 | 3.1 | 0 | 0 | 38.6 | 0.5 | 3.5 | 24 | 1.3 | 0.1 | 0.3 | 0 | 3.7 | 4.3 | 0 | 7.3 | 6.1 | 0.6 |
| *Pleuropterus* | 0.4 | 2.6 | 0.9 | 13.1 | 1.3 | 0.4 | 2.3 | 0.5 | 1 | 0.4 | 9.9 | 0.3 | 0 | 0.2 | 18.9 | 0 | 0 | 46.4 | 1.4 |
