# Supplementary material for: Plastome phylogenomics unveils an East Asian origin and climatic niche-driven radiation of the temperate tribe Polygoneae (Polygonaceae)
Source: Front Plant Sci. 2026 Mar 18;17:1792990. doi: 10.3389/fpls.2026.1792990 (PMC13038949; doi:10.3389/fpls.2026.1792990)
Supplement: Supplementary file 10 [file Table6.docx]

**Table S6.** Percent contribution of bioclimatic variables in MaxEnt ecological niche models across life forms and time periods (Last Interglacial, Mid-Holocene, Present).

| sample | vine-LIG | vine-MH | vine-Present | woody-LIG | woody-MH | woody-Present | herbal-LIG | herbal-MH | herbal-Present |
| --- | --- | --- | --- | --- | --- | --- | --- | --- | --- |
| bio_1% | 15.9 | 4.2 | 4.4 | 19.3 | 19.6 | 5.4 | 21.5 | 23.8 | 5.5 |
| bio_10% | 0.2 | 0 | 0 | 11.3 | 7.4 | 2.4 | 0.8 | 0.2 | 0 |
| bio_11% | 8 | 20.2 | 35.7 | 1.9 | 7.6 | 6.8 | 9.4 | 0.7 | 7.8 |
| bio_12% | 0.1 | 0.2 | 0.9 | 0.3 | 0.8 | 0.7 | 0.4 | 0.2 | 0.2 |
| bio_13% | 0.5 | 0 | 0 | 13.7 | 0.6 | 0.1 | 0 | 0 | 0.2 |
| bio_14% | 0.4 | 0 | 0.1 | 0.1 | 14.3 | 21.7 | 39.9 | 37.6 | 39.4 |
| bio_15% | 0.1 | 0.2 | 0.1 | 4 | 0.7 | 1.1 | 0.4 | 0.3 | 0.9 |
| bio_16% | 0 | 0.2 | 0.1 | 0.6 | 3.2 | 1.5 | 0.1 | 0.2 | 0 |
| bio_17% | 0.2 | 0 | 0 | 0.2 | 3.2 | 0.3 | 0.1 | 0 | 0 |
| bio_18% | 0.5 | 0.3 | 0.2 | 3.1 | 1.1 | 2.6 | 0.1 | 0.5 | 0.2 |
| bio_19% | 36.6 | 40.8 | 43 | 17 | 0.3 | 0.3 | 1 | 8.3 | 1.7 |
| bio_2% | 1 | 0.8 | 0.3 | 0.9 | 2 | 2.1 | 0.2 | 1.1 | 0.9 |
| bio_20% | 0.1 | 0.2 | 0 | 0.2 | 0.3 | 0.2 | 0 | 0 | 0 |
| bio_21% | 0 | 0 | 0 | 0.8 | 0.9 | 1.1 | 0.6 | 0.5 | 0.2 |
| bio_3% | 0.1 | 7.3 | 3.1 | 6.3 | 15.7 | 16.6 | 1.8 | 1.3 | 3.1 |
| bio_4% | 0.3 | 1 | 0.6 | 14.1 | 18.6 | 14.8 | 17.1 | 21.6 | 12.3 |
| bio_5% | 1.5 | 1.3 | 0.5 | 1.2 | 0.3 | 18.6 | 2.8 | 0.5 | 21.3 |
| bio_6% | 27 | 11.5 | 3.7 | 0.7 | 0.4 | 1.3 | 1.1 | 1.6 | 5.6 |
| bio_7% | 6.9 | 11.3 | 6.8 | 4.1 | 1.7 | 1.1 | 0.5 | 0.9 | 0.1 |
| bio_8% | 0.6 | 0.2 | 0.2 | 0 | 0.2 | 0.1 | 0.9 | 0.5 | 0.1 |
| bio_9% | 0 | 0.3 | 0.2 | 0 | 1.2 | 1.1 | 1.1 | 0.3 | 0.4 |
| SWR% | 0.1 | 0.2 | 0 | 0.8 | 0.9 | 1.1 | 0.6 | 0.5 | 0.2 |
| AWC% | 0 | 0 | 0 | 0.2 | 0.3 | 0.2 | 0 | 0 | 0 |
