# Supplementary material for: Plastome phylogenomics unveils an East Asian origin and climatic niche-driven radiation of the temperate tribe Polygoneae (Polygonaceae)
Source: Front Plant Sci. 2026 Mar 18;17:1792990. doi: 10.3389/fpls.2026.1792990 (PMC13038949; doi:10.3389/fpls.2026.1792990)
Supplement: Supplementary file 11 [file Table7.docx]

**Table S7.** Model performance evaluation (Area Under the Curve, AUC) for the Ecological Niche Models (ENMs).

|  | H-LIG | H-MH | H-P | W-LIG | W-MH | W-P | L-LIG | L-MH | L-P |
| --- | --- | --- | --- | --- | --- | --- | --- | --- | --- |
| AUC | 0.911 | 0.905 | 0.808 | 0.926 | 0.914 | 0.909 | 0.921 | 0.914 | 0.916 |
