# Supplementary material for: Plastome phylogenomics unveils an East Asian origin and climatic niche-driven radiation of the temperate tribe Polygoneae (Polygonaceae)
Source: Front Plant Sci. 2026 Mar 18;17:1792990. doi: 10.3389/fpls.2026.1792990 (PMC13038949; doi:10.3389/fpls.2026.1792990)
Supplement: Supplementary file 12 [file Table8.docx]

**Table S8.** Phylogenetic signal (Blomberg's K) estimates for eight morphological traits in Polygoneae.

| Trait | K statistic | p-value | Phylogenetic Signal | Evolutionary Mode |
| --- | --- | --- | --- | --- |
| Life history (annual/perennial) | 1.689 | 0.001 | Strong | Highly conserved |
| Pollen morphology | 1.471 | 0.001 | Strong | Highly conserved |
| Life form | 1.108 | 0.001 | Strong | Conserved (with global convergence) |
| Inflorescence structure | 0.286 | 0.001 | Weak | Labile / Convergent |
| Perianth merosity | 0.227 | 0.013 | Weak | Labile / Convergent |
| Fruit morphology | 0.171 | 0.026 | Weak | Labile / Convergent |
| Stigma | 0.122 | 0.057 | Not Significant | Evolutionarily labile |
| Stamen | 0.014 | 0.263 | Not Significant | Evolutionarily labile |
