# Supplementary material for: Plastome phylogenomics unveils an East Asian origin and climatic niche-driven radiation of the temperate tribe Polygoneae (Polygonaceae)
Source: Front Plant Sci. 2026 Mar 18;17:1792990. doi: 10.3389/fpls.2026.1792990 (PMC13038949; doi:10.3389/fpls.2026.1792990)
Supplement: Supplementary file 13 [file Table9.docx]

**Table S9.**

**Summary of principal components from the phylogenetic PCA of morphological traits in Polygoneae.**

| Principal Component | Standard Deviation | Proportion of Variance | Cumulative Proportion |
| --- | --- | --- | --- |
| PC1 | 30.258 | 86.808% | 86.808% |
| PC2 | 8.198 | 6.372% | 93.181% |
| PC3 | 4.819 | 2.202% | 95.383% |
| PC4 | 4.597 | 2.004% | 97.387% |
| PC5 | 4.173 | 1.651% | 99.038% |
| PC6 | 2.396 | 0.544% | 99.582% |
| PC7 | 1.718 | 0.280% | 99.862% |
| PC8 | 1.208 | 0.138% | 100.000% |

**Loadings of eight morphological traits on the principal components from the phylogenetic PCA.**

| Trait | PC1 | PC2 | PC3 | PC4 | PC5 | PC6 | PC7 | PC8 |
| --- | --- | --- | --- | --- | --- | --- | --- | --- |
| Fruit morphology | 0.206 | 0.036 | 0.528 | 0.759 | 0.012 | 0.310 | -0.071 | -0.024 |
| Life form | 0.022 | 0.061 | -0.795 | 0.364 | -0.469 | 0.051 | -0.059 | -0.066 |
| Perianth merosity | -0.011 | 0.044 | 0.461 | -0.404 | -0.785 | 0.073 | -0.017 | -0.012 |
| Inflorescence structure | -0.048 | -0.998 | -0.012 | 0.007 | -0.021 | 0.010 | -0.007 | 0.009 |
| Pollen morphology | -0.002 | 0.384 | -0.417 | 0.193 | -0.314 | 0.183 | -0.162 | 0.695 |
| Life year | -0.068 | -0.042 | -0.271 | 0.091 | -0.151 | 0.444 | 0.831 | 0.049 |
| Stigma morphology | -0.285 | 0.038 | -0.321 | -0.667 | 0.308 | 0.507 | -0.128 | -0.037 |
| Stamen morphology | -1.000 | 0.004 | 0.003 | 0.006 | -0.001 | -0.001 | ~0.000 | ~0.000 |
