# Supplementary material for: Plastome phylogenomics unveils an East Asian origin and climatic niche-driven radiation of the temperate tribe Polygoneae (Polygonaceae)
Source: Front Plant Sci. 2026 Mar 18;17:1792990. doi: 10.3389/fpls.2026.1792990 (PMC13038949; doi:10.3389/fpls.2026.1792990)
Supplement: Supplementary file 14 [file Table10.docx]

**Table S10. Plastid genes with signatures of positive or relaxed selection in Polygoneae.**

| **sample** | **value** |
| --- | --- |
| *psaI* | 1.395705521 |
| *accD* | 0.736667404 |
| *ycf2* | 0.647810219 |
| *matK* | 0.50697888 |
| *psbJ* | 0.497841727 |
| *rps12* | 0.392383525 |
| *psbF* | 0.367816092 |
| *rpoA* | 0.326009289 |
| *rps15* | 0.313144733 |
| *rps7* | 0.259206799 |
| *rpl32* | 0.247089263 |
| *psbL* | 0.235085647 |
| *atpF* | 0.232170423 |
| *psbK* | 0.22826087 |
| *rpl33* | 0.226324238 |
| *rpl20* | 0.213117047 |
| *rps16* | 0.203360703 |
| *psaJ* | 0.175596578 |
| *rps14* | 0.170169617 |
| *psbH* | 0.165145589 |
| *rpl2* | 0.16292735 |
| *ndhB* | 0.162548765 |
| *psbT* | 0.157234498 |
| *ndhK* | 0.155669321 |
| *ndhG* | 0.150238582 |
| *psbZ* | 0.139527559 |
| *ndhA* | 0.132006971 |
| *petA* | 0.130890052 |
| *infA* | 0.128556594 |
| *ycf4* | 0.119823686 |
| *ndhJ* | 0.116107931 |
| *rps4* | 0.115747536 |
| *petL* | 0.110680082 |
| *ndhD* | 0.105622326 |
| *clpP* | 0.101787843 |
| *psbN* | 0.100134409 |
| *rps3* | 0.099911471 |
| *rpoC1* | 0.097289268 |
| *rps18* | 0.096774194 |
| *atpE* | 0.096491228 |
| *rps8* | 0.095435072 |
| *rpoB* | 0.085526316 |
| *ndhE* | 0.08036036 |
| *rps11* | 0.080284285 |
| *atpI* | 0.078383978 |
| *atpA* | 0.070835452 |
| *psbI* | 0.060260744 |
| *psbB* | 0.057680051 |
| *rps19* | 0.05323264 |
| *ndhI* | 0.052923646 |
| *atpH* | 0.049851792 |
| *ndhC* | 0.048491014 |
| *psbD* | 0.034794711 |
| *psbE* | 0.033136632 |
| *rpl16* | 0.032791909 |
| *petB* | 0.030592105 |
| *psbC* | 0.02995671 |
| *psbM* | 0.022274115 |
| *rpl14* | 0.010947047 |
| *psaC* | 0.008283255 |
| *petD* | 0.004017793 |
