# Supplementary material for: Plastome phylogenomics unveils an East Asian origin and climatic niche-driven radiation of the temperate tribe Polygoneae (Polygonaceae)
Source: Front Plant Sci. 2026 Mar 18;17:1792990. doi: 10.3389/fpls.2026.1792990 (PMC13038949; doi:10.3389/fpls.2026.1792990)
Supplement: Supplementary file 15 [file Table11.docx]

**Table S11.** Pairwise niche overlap (Schoener's D) among studied Polygoneae lineages.

| **genus** | **sample1** | **sample2** | **sample3** | **sample4** | **sample5** | **sample6** | **sample7** | **sample8** | **sample9** | **sample10** |
| --- | --- | --- | --- | --- | --- | --- | --- | --- | --- | --- |
| sample1 | 0 | 0.383338 | 0.258195 | 0.102164 | 0.571491 | 0.625495 | 0.15685 | 0.096253 | 0.070521 | 0.327891 |
| sample2 | 0.383338 | 0 | 0.31932 | 0.143869 | 0.728309 | 0.37148 | 0.378301 | 0.369639 | 0.108742 | 0.113247 |
| sample3 | 0.258195 | 0.31932 | 0 | 0.114788 | 0.395417 | 0.52953 | 0.798657 | 0.823918 | 0.06675 | 0.084234 |
| sample4 | 0.102164 | 0.143869 | 0.114788 | 0 | 0.218744 | 0.119326 | 0.064574 | 0.155816 | 0.696851 | 0.040556 |
| sample5 | 0.571491 | 0.728309 | 0.395417 | 0.218744 | 0 | 0.358916 | 0.25732 | 0.192234 | 0.087382 | 0.071951 |
| sample6 | 0.625495 | 0.37148 | 0.52953 | 0.119326 | 0.358916 | 0 | 0.76251 | 0.708354 | 0.111014 | 0.153784 |
| sample7 | 0.15685 | 0.378301 | 0.798657 | 0.064574 | 0.25732 | 0.76251 | 0 | 0.942087 | 0.124688 | 0.07515 |
| sample8 | 0.096253 | 0.369639 | 0.823918 | 0.155816 | 0.192234 | 0.708354 | 0.942087 | 0 | 0.170876 | 0.050349 |
| sample9 | 0.070521 | 0.108742 | 0.06675 | 0.696851 | 0.087382 | 0.111014 | 0.124688 | 0.170876 | 0 | 0.171145 |
| sample10 | 0.327891 | 0.113247 | 0.084234 | 0.040556 | 0.071951 | 0.153784 | 0.07515 | 0.050349 | 0.171145 | 0 |
