# Supplementary material for: Plastome phylogenomics unveils an East Asian origin and climatic niche-driven radiation of the temperate tribe Polygoneae (Polygonaceae)
Source: Front Plant Sci. 2026 Mar 18;17:1792990. doi: 10.3389/fpls.2026.1792990 (PMC13038949; doi:10.3389/fpls.2026.1792990)
Supplement: Supplementary file 16 [file Table12.docx]

**Table S12.** Selected bioclimatic variables used for Ecological Niche Modeling (ENM) after multicollinearity screening.

|  | Herbaceous | Woody shrub | Liana |
| --- | --- | --- | --- |
| Present | bio1;bio2;bio3;bio4;bio7; bio10;bio15;bio16;bio18;bio19; | bio1;bio2;bio3;bio4;bio7; bio8;bio13;bio15;bio17;bio18;bio19; | bio1;bio2;bio3;bio4;bio5; bio7;bio10;bio13;bio15;bio17;bio18;bio19; |
| MH | bio1;bio2;bio4;bio5;bio7; bio10;bio13;bio15;bio17; | bio1;bio2;bio4;bio5;bio7; bio8;bio13;bio15;bio19; | bio1;bio2;bio4;bio5;bio7; bio10;bio13;bio14;bio15;bio17; |
| LIG | bio1;bio2;bio4;bio7;bio10; bio13;bio15;bio18; | bio1;bio2;bio4;bio7;bio9; bio14;bio15;bio17; | bio1;bio3;bio4;bio7; bio9;bio15;bio16;bio17; |
